# A stromal inflammasome Ras-safeguard against Myc driven lymphomagenesis

**DOI:** 10.1038/s41590-024-02028-z

**Published:** 2025-01-02

**Authors:** Andrew Kent, Kristel Joy Yee Mon, Zachary Hutchins, Gregory Putzel, Dmitry Zhigarev, Alexander Grier, Baosen Jia, Roderik M. Kortlever, Gaetan Barbet, Gerard I. Evan, J. Magarian Blander

**Affiliations:** 1 Division of Hematology, University of Colorado School of Medicine, Aurora, CO, USA; 2 Jill Roberts Institute for Research in Inflammatory Bowel Disease, Weill Cornell Medicine, Cornell University, New York, NY, USA; 3 Joan and Sanford I. Weill Department of Medicine, Weill Cornell Medicine, Cornell University, New York, NY, USA; 4 Department of Microbiology and Immunology, Weill Cornell Medicine, Cornell University, New York, NY, USA; 5 Sandra and Edward Meyer Cancer Center, Weill Cornell Medicine, Cornell University, New York, NY, USA; 6 The Francis Crick Institute, London, UK; 7 Kings College London, London, UK; 8 Immunology and Microbial Pathogenesis Program, Weill Cornell and Sloan Kettering Institute Graduate School of Medical Sciences, New York, NY, USA

## Abstract

The inflammasome plays multifaceted roles in cancer, but less is known about its function during premalignancy upon initial cell transformation. We report a homeostatic function of the inflammasome in suppressing malignant transformation through Ras inhibition. We identified increased hematopoietic stem cell (HSC) proliferation within the bone marrow of inflammasome-deficient mice. HSCs within an inflammasome-deficient stroma expressed a Ras-signature associated with increased Ras pathway and cancer-related transcripts and heightened levels of cytokine, chemokine, and growth-factor receptors. Stromal inflammasome deficiency established a poised Ras-dependent mitogenic state within HSCs, which fueled progeny B cell lymphomagenesis upon Myc deregulation in a spontaneous model of B cell lymphoma, and shortened its premalignant stage leading to faster onset of malignancy. Thus, the stromal inflammasome preserves tissue balance by restraining Ras to disrupt the most common oncogenic Myc-Ras cooperation and establish a natural defense against transition to malignancy. These findings should inform preventative therapies against hematological malignancies.

Innate immune surveillance against the progression of precancerous lesions to invasive cancer within a tissue is poorly understood. Little research has focused on the early phases of cancer because appropriate models that recapitulate the evolution of cancer from premalignancy to malignancy are limited and technically challenging to work with. Furthermore, clinical presentation occurs when malignant disease is already established, thus human data relevant to immune regulation of premalignancy is scarce^1^. The inflammasome is a critical pathway of the innate immune system that initiates an inflammatory response to various dangers^2^. It is composed of multiprotein complexes that play a pivotal role in host defense against pathogens and in response to cellular damage or stress. Prior studies had established involvement of the inflammasome, especially the NLRP3 inflammasome, in the advanced stages of tumorigenesis^3,4,5,6,7,8^. The role of inflammasomes in premalignancy, when transformed cells first appear in tissue, is poorly understood.

Using the transgenic *Eμ-myc* mouse model of B cell lymphoma, where c-*Myc* deregulation in pre-B cells models spontaneous B cell lymphoma^9,10^, we found that the inflammasome in the bone marrow stroma orchestrated constraint of the Ras pathway *in trans* within hematopoietic stem cells (HSCs) and controlled turnover of stem and progenitor cells in the transformed compartment. Inflammasome deficiency in *Eμ-myc* mice shortened the characteristic extended premalignant stage in this model and led to faster onset of malignancy. We noted increased proliferation of early stem and progenitor cells in premalignant inflammasome-deficient *Eμ-myc* mice, and even during homeostasis in non-transgenic inflammasome-deficient mice. Inflammasome deficiency was associated with a Ras activation signature in HSCs and their heightened expression of cytokine, chemokine, and growth factor receptors. Stromal control of HSC homeostasis involved both a soluble trans-acting factor and stroma-HSC proximity and depended on an NLRP3-ASC-caspase-1-gasdermin D inflammasome module. Inhibiting caspase function in the stroma mirrored these effects, while Ras inhibition in HSCs was associated with their reversal. Our findings indicate that the stromal inflammasome maintains tissue homeostasis by restraining Ras to undermine the most prevalent oncogenic cooperation between Myc and Ras^11,12,13^ and form an innate barrier against premalignant-to-malignant transition.

## Transformed cell expansion upon inflammasome impairment

**Extended Data Figure 1:**
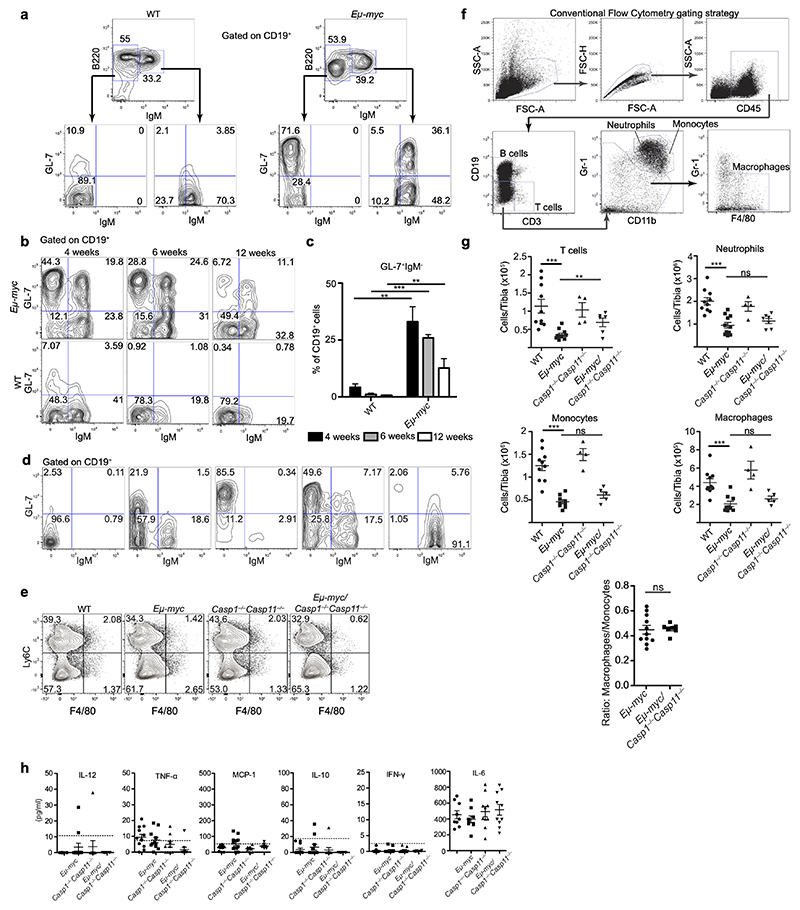


To define the effects of the inflammasome on premalignancy, we chose the *Eμ-myc* mouse model because of its spontaneous nature and characteristic prolonged premalignant stage. In *Eμ* mice, the proto-oncogene *c-Myc* is expressed under the *Eμ-*heavy chain enhancer to model genetic Myc rearrangement in several aggressive B cell lymphomas^14^. We crossed *Eμ-myc* mice to mice deficient in both caspases 1 and 11 (*Casp1*^*−/−*^*Casp11*^*129mt/129/mt*^ hereafter referred to as *Casp1*^*−/−*^*Casp11*^*−/−*^), which are essential for all known effector functions of canonical and non-canonical inflammasomes. Premalignant *Eμ-myc* mice exhibit expansion of a B220^lo^CD19^+^ B cell progenitor population that we found to also be GL-7^+^IgM^*−*15,16^. These GL-7^+^IgM^–^ pre-B cells are largely absent in the circulation of non-transgenic wild-type (WT) syngeneic C57BL/6J mice (57.35+/-14.28% vs. 6.26+/-2.31%, Extended Data Fig. 1a)^17^. The B220^hi^ population was comprised of GL-7^+^IgM^+^ late pre-B cells, which were also increased in percentage in premalignant *Eμ-myc* mice compared to WT controls, while GL-7^−^IgM^+^ immature B cells were lower in frequency compared to WT mice (Extended Data Fig. 1a). Consistently, the percentage of circulating GL-7^+^IgM^–^ cells decreased dramatically after 4 weeks of age (Extended Data Fig. 1b,c). By contrast, circulating CD19^+^ cells at the malignant stage of disease exhibited varying surface phenotypes of GL-7 and IgM expression (Extended Data Fig. 1d). For consistency, we used 4-week old mice for all subsequent analyses, and GL-7 as a marker to track the premalignant population.

**Figure 1:**
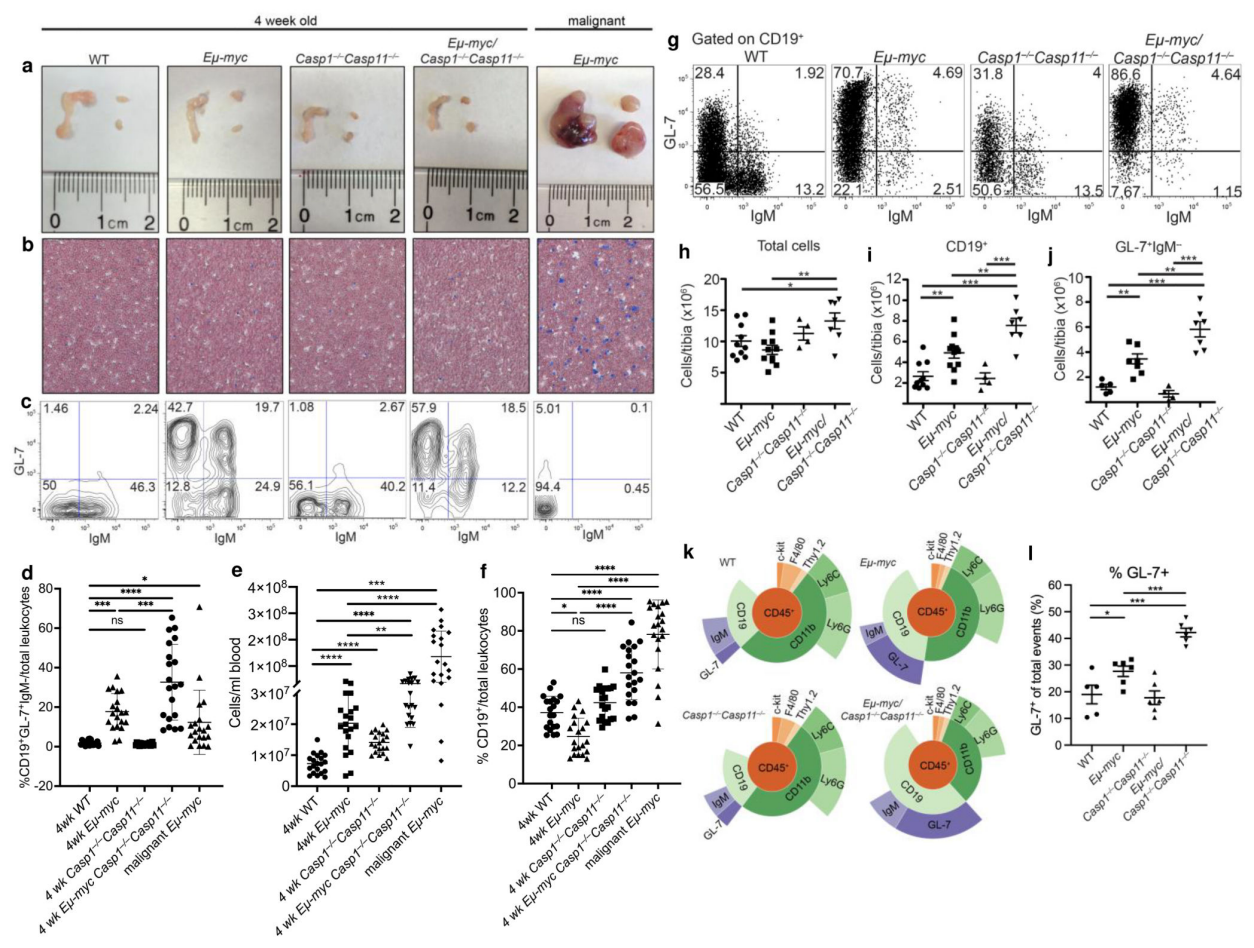
Inflammasome impairment expands circulating and bone marrow B cell progenitors in premalignant *Eμ-myc* mice. **a-c**, Representative images and plots from 4-week-old pre-malignant mice of each genotype and malignant *Eμ-myc* mice. **a**, Gross pathology of mesenteric, axial, and inguinal lymph nodes. **b**, Giemsa-stained peripheral blood smears showing degrees of leukocytosis. **c**. Flow cytometry of peripheral blood pre-gated on CD19^+^ cells, stained with the B cell markers IgM and GL-7. Data in **a-c** represent at least 10 mice/genotype. **d-f**, Quantification of CD19^+^GL-7^+^IgM^–^ frequency (**d**), total leukocyte counts (**e**), and frequency of CD19^+^ cells (**f**) in the blood. Data in **d-f** represent at least 10 mice/genotype. **g**, Representative flow cytometry of bone marrow CD19^+^ cells stained for GL-7 and IgM. **h-j**, Quantification of total bone marrow (**h**), and absolute numbers of CD19^+^ (**i**), and GL-7^+^IgM^−^ (**j**) cells from conventional flow gating strategy. Data in h-j represent at least 4 mice/genotype. **g**, Cytokine concentration measurements on bone marrow supernatants determined by cytometric bead array. Data represent four independent experiments, at least eight mice per group. **k**, Representative CyTOF Sunburst diagrams where the % of the circumference for a given cell gate corresponds to the frequency of that group out of the total CD45^+^ cells in the bone marrow. Data in (**k**) represent 6 mice/genotype. **l**, Quantification of GL-7^+^ cells out of total cells in the bone marrow from our CyTOF data (6 mice per group). Each symbol represents an individual mouse. Data represent at least three independent experiments. p^*^≤0.05, p**≤0.01, p***≤0.001, p****≤0.0001 calculated by two-tailed student’s t-test or 1-Way ANOVA with Sidak’s posttest. All quantified data are presented as mean values +/- SEM.

We focused on the premalignant stage of disease using malignant stage *Eμ-myc* mice as a reference. At four weeks of age, neither *Eμ-myc Casp1*^*−/−*^*Casp11*^*−/−*^ nor *Eμ-myc* mice showed any signs of lymphadenopathy (Fig. 1a) or appearance in the blood of large blast-like cells (Fig. 1b), features characteristic of the malignant stages of disease in *Eμ-myc* mice (Fig. 1a,b malignant panels and Extended Data Fig. 1d). Despite having no gross signs of pathology, we noted significantly increased percentages of circulating CD19^+^GL-7^+^IgM^−^ pre-B cells, as well as total cells, in premalignant *Eμ-myc Casp1*^*−/−*^*Casp11*^*−/−*^ mice compared to *Eμ-myc* or WT mice (Fig. 1c and quantified in Fig. 1d, e). Opposite to *Eμ-myc Casp1*^*−/−*^*Casp11*^*−/−*^ mice, the CD19^+^ B cell frequency in the peripheral blood of *Eμ-myc* mice during pre-malignancy was significantly decreased compared to WT mice, but almost 100% of leukocytes in *Eμ-myc* mice were CD19^+^ leukemic cells after malignant transformation (Fig. 1f). Collectively, these data demonstrate an expansion of the transformed B cell compartment, and specifically at the progenitor stage, within the circulation in premalignant *Eμ-myc Casp1*^*−/−*^*Casp11*^*−/−*^ mice.

Mirroring our findings in the circulation, we observed an increased frequency of GL-7^+^IgM^−^ pre-B cells in the bone marrow of pre-malignant *Eμ-myc* and *Eμ-myc Casp1*^*−/−*^*Casp11*^*−/−*^ mice (Fig. 1g). The total numbers of bone marrow cells (Fig. 1h) and bone marrow CD19^+^ cells (Fig. 1i) were significantly increased in *Eμ-myc Casp1*^*−/−*^*Casp11*^*−/−*^ compared to *Eμ-myc* and WT mice. GL-7^+^IgM^−^ pre-B cells, comprising the largest fraction within the CD19^+^ compartment, were also significantly increased in number in *Eμ-myc Casp1*^*−/−*^*Casp11*^*−/−*^ compared to *Eμ-myc* mice (Fig. 1j). This cellular increase was specific to *Casp1* and *Casp11* deficiency on the *Eμ-myc* background because *Casp1*^*−/−*^*Casp11*^*−/−*^ littermates did not have increased cellularity compared to WT mice (Fig. 1h–j).

Inflammatory mediators in the bone marrow microenvironment are known to impact the size of the transformed compartment and modulate leukemic progression^18^. Checking for signs of inflammation in the bone marrow of premalignant mice, we observed no difference in the distribution of Ly6C and F4/80 staining in myeloid cells in *Eμ-myc Casp1*^*−/−*^*Casp11*^*−/−*^ compared to *Eμ-myc* mice (Extended Data Fig. 1e). A flow cytometry gating strategy to further delineate T cells, neutrophils and myeloid cells in the bone marrow (Extended Data Fig. 1f) showed a significant decrease in the absolute numbers of T cells, neutrophils, monocytes, and macrophages in both premalignant *Eμ-myc* and *Eμ-myc Casp1*^*−/−*^*Casp11*^*−/−*^ mice compared to their non-transgenic controls, and no difference in the ratio of monocytes to macrophages that would have suggested inflammation-induced infiltration of inflammatory Ly6C^+^ monocytes (Extended Data Fig. 1g). Furthermore, we were unable to detect any appreciable changes in an array of inflammatory cytokines within the bone marrow, including IL-12, TNF and IL-6 (Extended Data Fig. 1h).

**Extended Data Figure 2:**
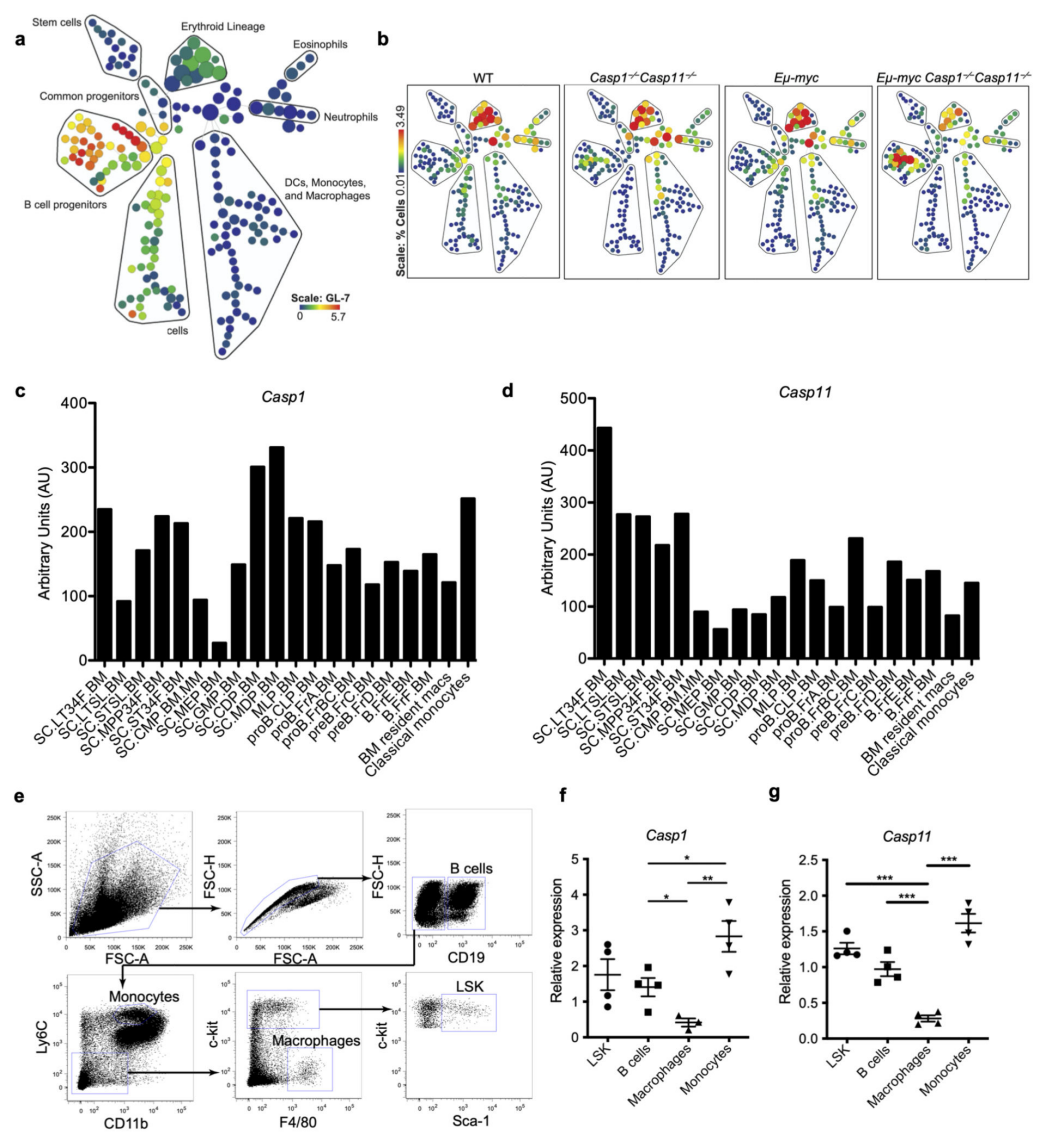


We confirmed our conventional flow cytometry findings using a CyTOF mass cytometry approach^19^. Using a panel of 34 CyTOF markers, we delineated the hematopoietic tree from the earliest stem cells through the lymphoid, myeloid, and erythroid lineages, with particular resolution in the B cell populations (Extended Data Fig. 2a,b and Supplementary Table 1). Representative Sunburst visualizations^20^ demonstrated an increased frequency of B lineage cells at the expense of the myeloid compartment in *Eμ-myc* mice, which was more pronounced in *Eμ-myc Casp1*^*−/−*^*Casp11*^*−/−*^ mice (Fig. 1k). Quantification revealed a significant increase of GL-7^+^ B cells in *Eμ-myc Casp1*^*−/−*^*Casp11*^*−/−*^ bone marrow compared to WT or *Eμ-myc* mice (Fig. 1l). Significantly increased numbers of transformed CD19^+^GL-7^+^IgM^−^ pre-B cells in the circulation and the bone marrow of inflammasome deficient *Eμ-myc* mice were concordant with indications of disease acceleration and dissemination^10,21^.

**Extended Data Figure 3:**
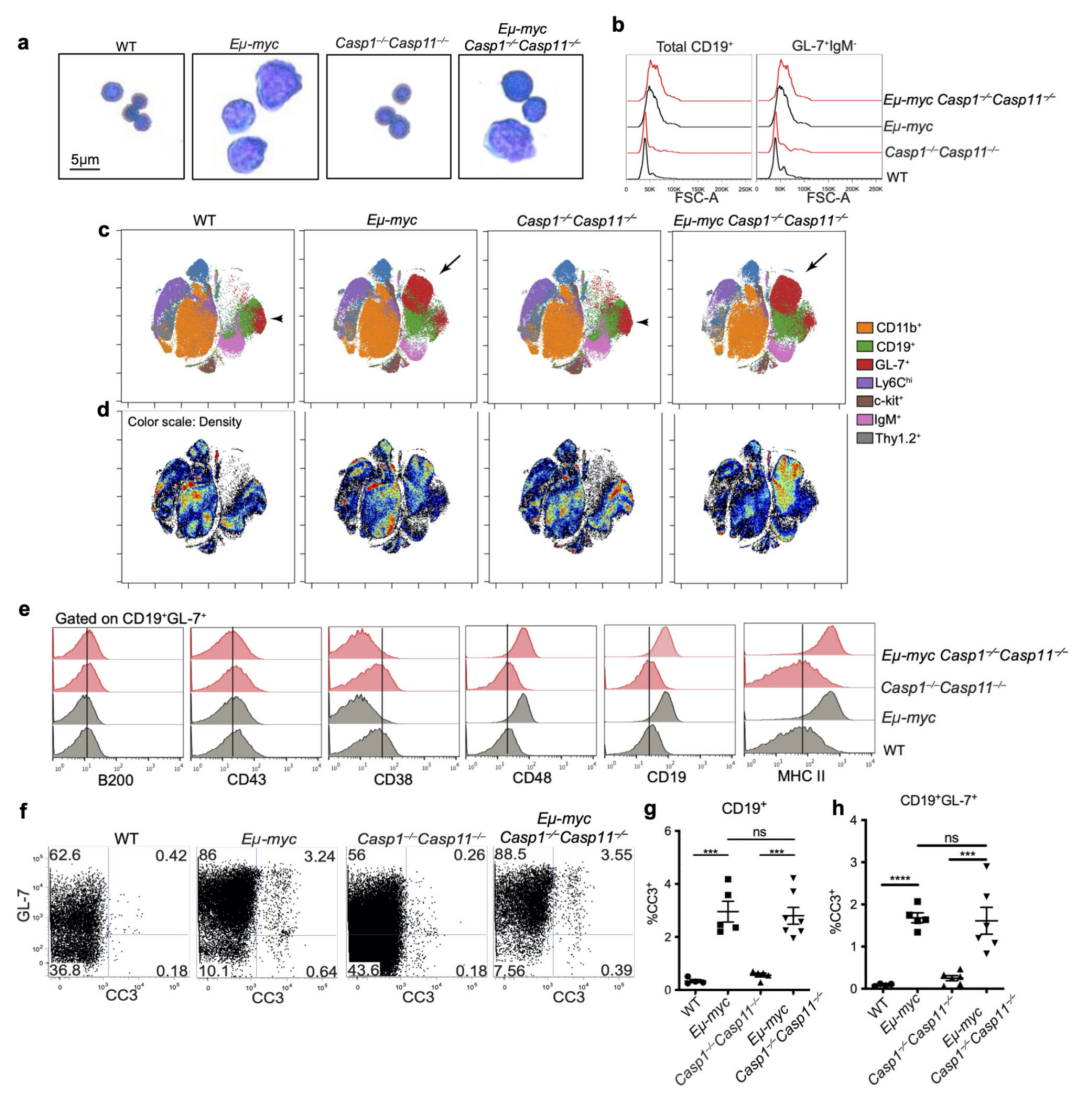


Using the Immunological Genome (ImmGen) database^22^ (Extended Data Fig. 2c,d) as well as a flow cytometry sorting strategy followed by quantitative polymerase chain reaction (qPCR) (Extended Data Fig. 2e-g), we found that many hematopoietic cell types in the bone marrow expressed *Casp1* and *Casp11* including B cells and their progenitors. B cell intrinsic inflammatory caspase-mediated effects could have explained the increase in numbers of B lineage cells in *Eμ-myc Casp1*^*−/−*^*Casp11*^*−/−*^ mice. Transformed cells were much larger regardless of *Casp1* and *Casp11* deficiency (Extended Data Fig. 3a,b). CyTOF data analysis using viSNE plots^23^ showed that the density (Extended Data Fig. 3c) but not clustering position (Extended Data Fig. 3d) of these cells differed between *Eμ-myc* and *Eμ-myc Casp1*^*−/−*^*Casp11*^*−/−*^ indicating that *Eμ-myc Casp1*^*−/−*^*Casp11*^*−/−*^ mice had the same aberrant populations of cells as *Eμ-myc* mice, the difference lying in their frequency rather than phenotype. Regardless of inflammasome impairment, transformed cells expressed comparable levels of B220 and CD43, lower levels of CD38, and higher levels of CD19 and MHC-II than their non-transformed counterparts (Extended Data Fig. 3e). Lastly, similar frequencies of CD19^+^cells and CD19^+^GL-7^+^ cells that were positive for cleaved caspase-3, a marker for apoptosis, were present in *Eμ-myc* and *Eμ-myc Casp1*^*−/−*^*Casp11*^*−/−*^ bone marrow compared to control mice (Extended Data Fig. 3f-h). Collectively, the data argued against alterations in pre-B cell intrinsic phenotype, survival mechanisms, or inflammation being the cause of the increased transformed cell numbers in premalignant *Eμ-myc Casp1*^*−/−*^*Casp11*^*−/−*^ mice.

## Stromal inflammasome impairment shortens premalignancy

**Figure 2:**
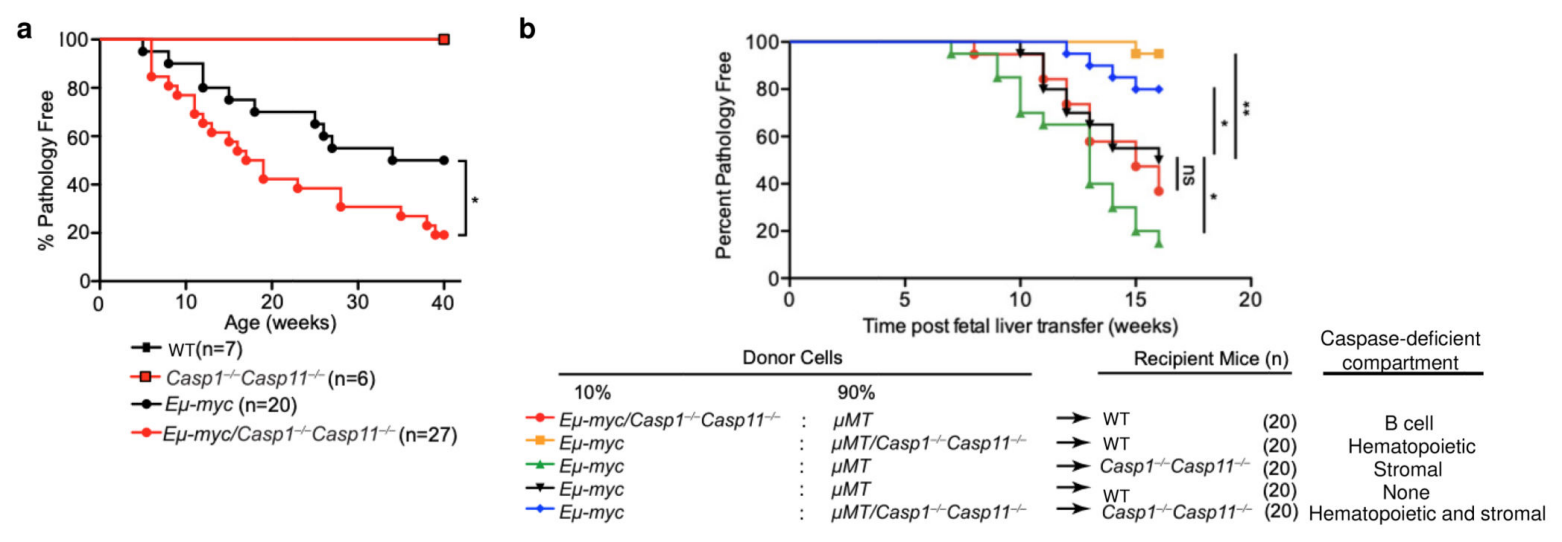
Stromal inflammasome deficiency accelerates malignant disease onset in *Eμ-myc mice*. **a**, Kaplan-Meier curves of cohorts of mice of each genotype indicating age at first pathology. **b**, Kaplan-Meier curves of mixed fetal liver chimeras. In a 9:1 ratio, either *μ*MT or *μ*MT/ *Casp1*^*−/−*^*Casp11*^*−/−*^ bone marrow mixed with either *Eμ-myc* or *Eμ-myc Casp1*^*−/−*^*Casp11*^*−/−*^ fetal liver cells were used to reconstitute WT and *Casp1*^*−/−*^*Casp11*^*−/−*^ recipient mice, as indicated. In resultant chimeras, *Casp1* and *Casp11* deficiency was isolated to either the B cell compartment (red), the non-B cell hematopoietic compartment (yellow), the stromal compartment (green), or both the stromal and non-B cell hematopoietic compartments (blue). Control chimeras with no *Casp1* or *Casp11* deficiency shown as black. **a**,**b**, Mouse numbers in each group indicated in parentheses. p*≤0.05, p**≤0.01, calculated by Mantel-Cox Log-Rank test.

We next monitored cohorts of *Eμ-myc* and *Eμ-myc Casp1*^*−/−*^*Casp11*^*−/−*^ mice for disease free survival. Onset of the malignant stage of disease was significantly faster in *Eμ-myc Casp1*^*−/−*^*Casp11*^*−/−*^ compared to *Eμ-myc* mice (Fig. 2a). After 40 weeks, 50% of *Eμ-myc* mice were still pathology free, while only 19.2% of *Eμ-myc Casp1*^*−/−*^*Casp11*^*−/−*^ mice were pathology free. To narrow down the compartment in which inflammasome activity influences lymphoma progression, we performed mixed fetal liver chimera experiments reconstituting lethally irradiated recipient WT or *Casp1*^*−/−*^*Casp11*^*−/−*^ with a 9:1 ratio of B cell deficient *μ*MT WT or *μ*MT/*Casp1*^*−/−*^*Casp11*^*−/−*^ and *Eμ-myc* or *Eμ-myc/Casp1*^*−/−*^*Casp11*^*−/−*^ bone marrows as depicted in Fig. 2b. Accordingly, *Casp1/Casp11* deficiency was either limited to transformed B cells, non-B cell hematopoietic cells, and/or the recipient mouse stroma. We monitored pathology-free survival of these cohorts over time, and observed that inflammasome impairment in the stromal compartment (green) phenocopied the reduced pathology-free survival observed in *Eμ-myc Casp1*^*−/−*^*Casp11*^*−/−*^ (Fig. 2b). Inflammasome deficiency within the B cell compartment (red) had no impact on cancer kinetics compared to all inflammasome sufficient chimeras (black) (Fig. 2b), concordant with the lack of phenotypic changes we had noted within B cells upon intrinsic inflammasome impairment. Because *Casp1/Casp11* deficiency in the stromal compartment recapitulated the findings in our non-chimeric *Eμ-myc Casp1*^*−/−*^*Casp11*^*−/−*^ mice, these data indicated that inflammasome impairment in the stroma accounted for accelerated tumorigenesis.

## The inflammasome restricts bone marrow HSC proliferation

**Figure 3:**
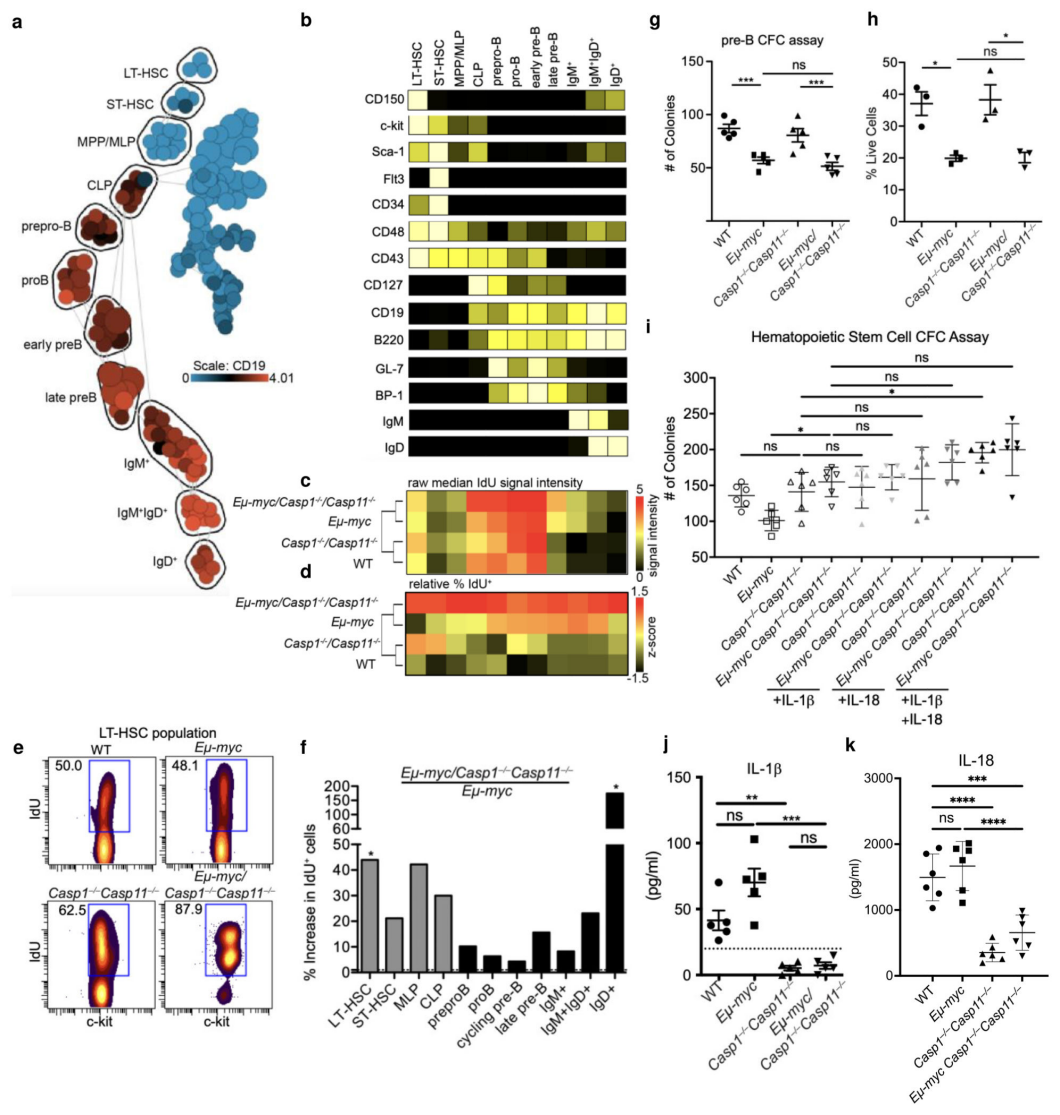
Inflammasome impairment accelerates bone marrow stem and B cell progenitor proliferation in *Eμ-myc mice*. **a**, Representative SPADE tree analysis of CyTOF data depicting the gating strategy for the B cell lineage, from hematopoietic stem cell (HSC) through fully differentiated B cell (LT-HSC = long-term HSC; ST-HSC = short-term HSC; MPP/MLP = multipotent progenitor/multilineage progenitor; CLP = common lymphoid progenitor). **b**, Representative heatmap of the B cell lineage from the gating strategy in (**a**) showing relative staining intensity of the indicated markers for each population in the bone marrows of 4-week-old mice. **c**, Heatmap of the raw % IdU positive cells for each population for each genotype (3 mice per group). **d**, Heatmap of relative %IdU positive cells per population, normalized within each population column across the four genotypes (3 mice per group). **e**, Representative bidirectional plots of CyTOF IdU staining within the LT-HSC population from the bone marrow of each indicated genotype. **f**, Bar chart depicting the % increase in IdU^+^ cell frequency within each indicated population comparing *Eμ-myc Casp1*^*−/−*^*Casp11*^*−/−*^ to *Eμ-myc*. Significance determined by student t-test of IdU^+^ cell frequency within each population (3 mice per group). **g**, Pre-B cell colony formation (CFC) assay using bone marrow from the indicated genotypes, 5 biological replicates/genotype. **h**, Cell viability in the pre-B CFC assays from (**g**) as determined by Annexin-V^+^7-AAD^+^ staining using flow cytometry, 3 biological replicates/genotype. **i**, HSC colony formation in complete methylcellulose media without Epo with plated bone marrow from the indicated genotypes and cytokine treatment groups, n = a single 2cm x 2cm area of formed colony counts for each of the 5 biological replicates per genotype. **j**, IL-1β concentrations in bone marrow supernatants determined by cytometric bead array, 5 biological replicates/genotype. **k**, IL-18 concentrations in bone marrow supernatants determined by ELISA, 6 biological replicates/genotype p*≤0.05, p**≤0.01, p***≤0.001, calculated by two-tailed unpaired student’s T-test. All quantified data are presented as mean values +/- SEM.

Using our CyTOF data, we outlined spanning-tree progression analysis of density-normalized events (SPADE)^24^ groups corresponding to eleven stem cell, progenitor, and B cell populations within the bone marrow (Fig. 3a,b). Using an *in vivo* 5-Iodo-2’-deoxyuridine (IdU) pulse to measure cell turnover, we observed higher frequencies of IdU^+^ cells for every population in *Eμ-myc Casp1*^*−/−*^*Casp11*^*−/−*^ bone marrow relative to the other genotypes (Fig. 3c,d). The largest difference in IdU incorporation in the bone marrow between *Eμ-myc* and *Eμ-myc Casp1*^*−/−*^*Casp11*^*−/−*^ mice was in the earliest HSC population (LT-HSC, Fig. 3e,f), as well as differentiated IgD^+^ B cells (Fig. 3f). We also detected increased IdU incorporation in the earliest stem cells but not normal mature IgD^+^ B cells, in control *Casp1*^*−/−*^*Casp11*^*−/−*^ compared to WT mice (Fig. 3c,d), indicative of inflammasome-dependent regulation of stem cell turnover even in the absence of transformation.

Comparing *Eμ-myc* and *Eμ-myc Casp1*^*−/−*^*Casp11*^*−/−*^ mice, there was no significant difference in IdU incorporation in the prepro-B to late pre-B cell stages when the Myc oncogene is expressed (Fig. 3d,f), and no difference in pre-B colony forming ability between *Eμ-myc* and *Eμ-myc Casp1*^*−/−*^*Casp11*^*−/−*^ bone marrow (Fig. 3g), concordant with a lack of an intrinsic role for caspases 1 and 11 within these cells. The Myc transformed bone marrows formed fewer B cell colonies *ex vivo* than their non-transformed counterparts (Fig. 3g) likely due to decreased cell viability (Fig. 3h). Cell viability in the *ex vivo* pre-B colony formation assays was comparable between *Eμ-myc* and *Eμ-myc Casp1*^*−/−*^*Casp11*^*−/−*^ cells (Fig. 3h). In contrast, sorted *Eμ-myc Casp1*^*−/−*^*Casp11*^*−/−*^ lineage^*−*^Sca-1^+^c-kit^+^ (LSK) stem cells formed significantly more colonies *ex vivo* than *Eμ-myc* stem cells (Fig. 3i), likely due to their higher proliferation rate (Fig. 3c–f). While detectable IL-1β, reported to accelerate HSC division^25,26^, and IL-18, shown to suppress short term bone marrow progenitors^27,28^, were not statistically different between WT and *Eμ-myc* mice, their levels were significantly reduced in both *Casp1*^*−/−*^*Casp11*^*−/−*^ and *Eμ-myc Casp1*^*−/−*^*Casp11*^*−/−*^ bone marrows compared to their WT counterparts (Fig. 3j,k). Exogenous recombinant IL-1β and IL-18, alone or in combination, had no statistically significant impact on *Eμ-myc Casp1*^*−/−*^*Casp11*^*−/−*^ derived HSC colony formation (Fig. 3i). Collectively, these data revealed that the accelerated onset of malignant disease upon inflammasome impairment in *Eμ-myc Casp1*^*−/−*^*Casp11*^*−/−*^ was associated with an increase in HSC and progenitor B cell proliferation during pre-malignancy.

## Inflammasome impairment modulates the HSC transcriptome

**Extended Data Figure 4:**
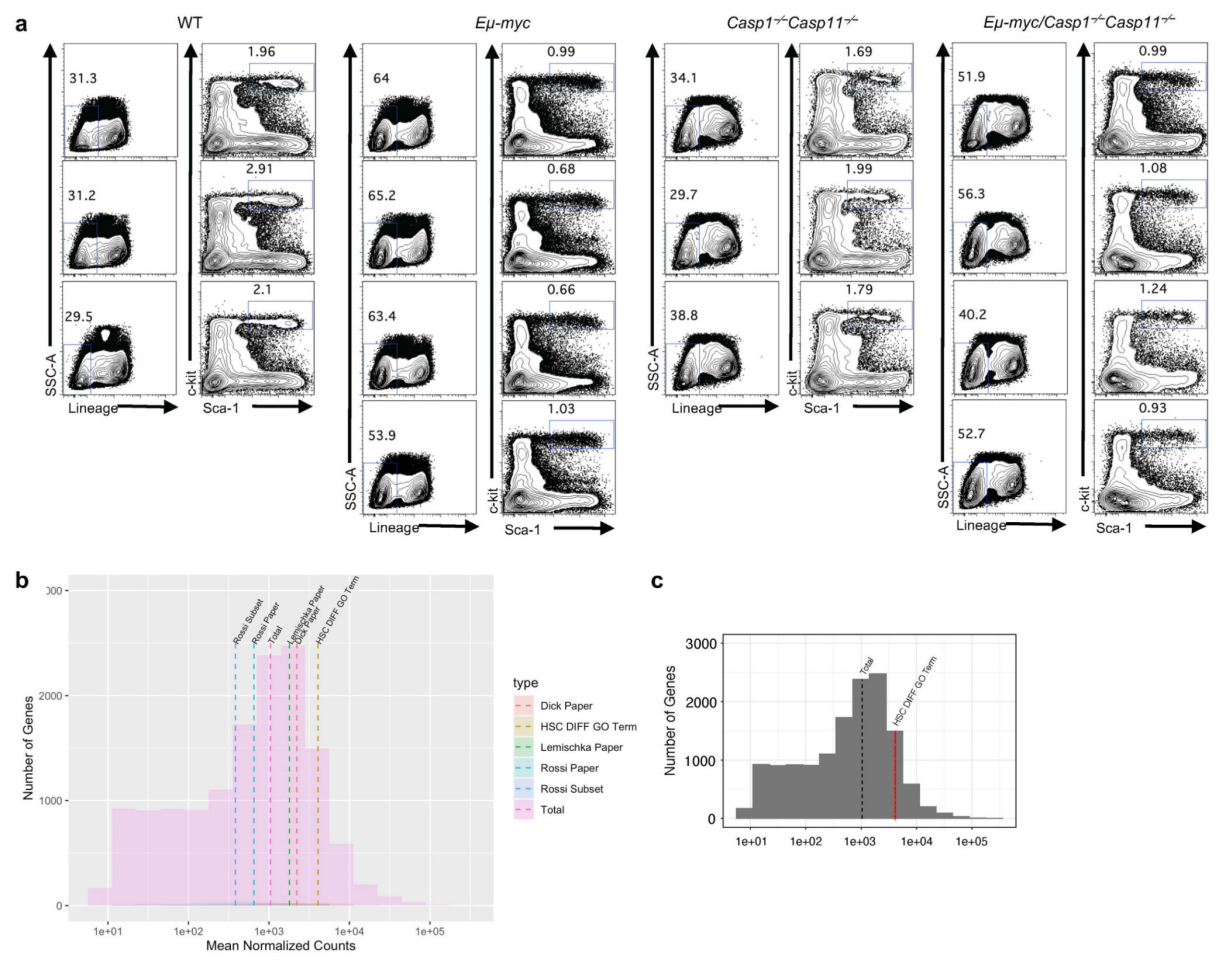


**Figure 4:**
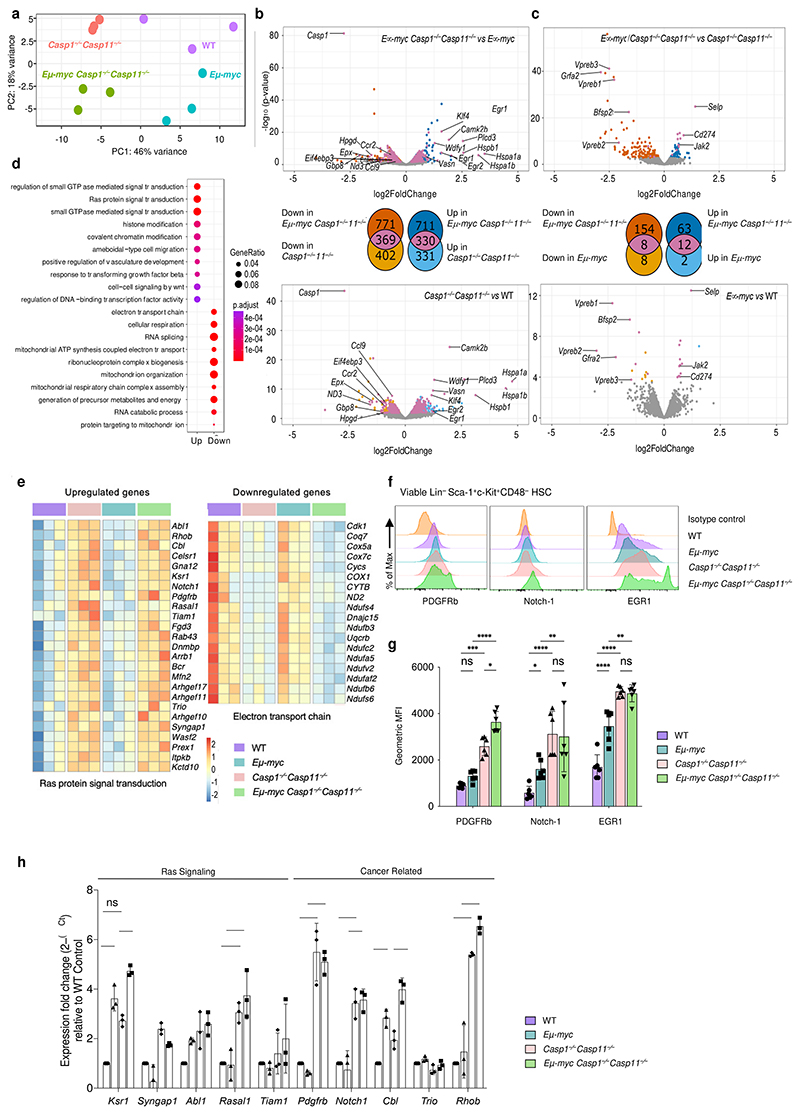
A heightened HSC Ras signaling signature upon inflammasome deficiency. **a**, Principal components analysis (PCA) plot of RNAseq-derived transcriptomes of sorted HSCs. **b**, Volcano plots showing differential expression due to *Casp1*^*−/−*^*Casp11*^*−/−*^ in HSCs of *Eμ-myc* (top) and wild type (bottom) mice. Positive log fold changes indicate higher expression in *Casp1*^*−/−*^*Casp11*^*−/−*^. Points representing significantly differentially expressed genes (adjusted p < 0.1) are colored according to the Venn diagrams, which indicate the numbers of differentially expressed genes shared in the wild type and *Eμ-myc* backgrounds. **c**, Volcano plots showing differential expression due to *Eμ-myc* in *Casp1*^*−/−*^*Casp11*^*−/−*^ (top) and wild type (bottom) mice. Differentially expressed genes are colored according to the Venn diagrams, which show differentially expressed genes shared in the *Casp1*^*−/−*^*Casp11*^*−/−*^ and wild type backgrounds. **d**, Gene Ontology (GO) biological process terms most significantly enriched among genes differentially expressed due to caspase 1 and caspase 11 deficiency in both *Eμ-myc* and wild type backgrounds (common genes indicated in purple in (**b**)). (b,d) Enrichment p values were corrected using the method of Benjamini and Hochberg. **e**, Heatmaps showing normalized expression levels of differentially expressed genes from selected enriched GO terms. Genes shown are differentially regulated by *Casp1*^*−/−*^*Casp11*^*−/−*^ in both *Eμ-myc* and wild type backgrounds. Color scale indicates row z score of normalized read counts. **f**, Representative histograms of normalized Ras target expression in Lin^–^Sca1^+^c-Kit^+^CD48^–^ HSC from bone marrow of adult male *Wt, Casp1*^*−/−*^*Casp11*^*−/−*^, *Eμ-myc* and *Eμ-myc Casp1*^*−/−*^*Casp11*^*−/−*^ mice. **g**, Quantification of gMFI of (**f**); n=6 biological replicates per genotype; p*≤0.05, p**≤0.01, p***≤0.001, p****≤0.0001; 2 WAY Anova. **h**, Quantitative real time PCR showing fold change expression of Ras signaling associated, and cancer related genes derived from RNA-Seq data relative to WT (normalized to β2M at n=1) of enriched HSCs from bone marrow of adult male WT, *Casp1*^*−/−*^*Casp11*^*−/−*^, *Eμ-myc* and *Eμ-myc Casp1*^*−/−*^*Casp11*^*−/−*^ mice; n=3 biological replicates per genotype; p*≤0.05, p**≤0.01, p***≤0.001, p****≤0.0001; 2 WAY Anova. All data are presented as mean values +/-SD.

We next conducted RNA-sequencing on isolated LSK HSCs from premalignant *Eμ-myc* and *Eμ-myc Casp1*^*−/−*^*Casp11*^*−/−*^ and their non-transgenic WT and *Casp1*^*−/−*^*Casp11*^*−/−*^ mice counterparts (Extended Data Fig. 4a). We found 322 genes with enriched expression in sorted LSK HSCs compared to all other hematopoietic cell types in the Immunological Genome (ImmGen) database^22^ (Supplementary Data 1). Genes reported to be restricted to HSCs^29,30,31^ were highly expressed (Extended Data Fig. 4b) with highest enrichment in HSC signature genes as represented in the Hematopoietic Stem Cell Differentiation gene ontology (GO) biological process (Extended Data Fig. 4c). Principal components analysis (PCA) showed that all four HSC groups were transcriptionally distinct, with the effect of caspase-1/11 deficiency being stronger than the *Myc* transgene at this premalignant stage (Fig. 4a). The transcriptional effect of caspase-1/11 deficiency on HSCs was broadly similar between non-transgenic and transgenic *Eμ-myc* backgrounds, with hundreds of differentially expressed genes associated with caspase-1 and 11 deficiency, many of which were common to the non-transgenic and *Eμ-myc* backgrounds (Fig. 4b). We found fewer differentially expressed genes associated with the *Eμ-myc* background irrespective of *Casp1/Casp11* deficiency (Fig. 4c).

**Extended Data Figure 5:**
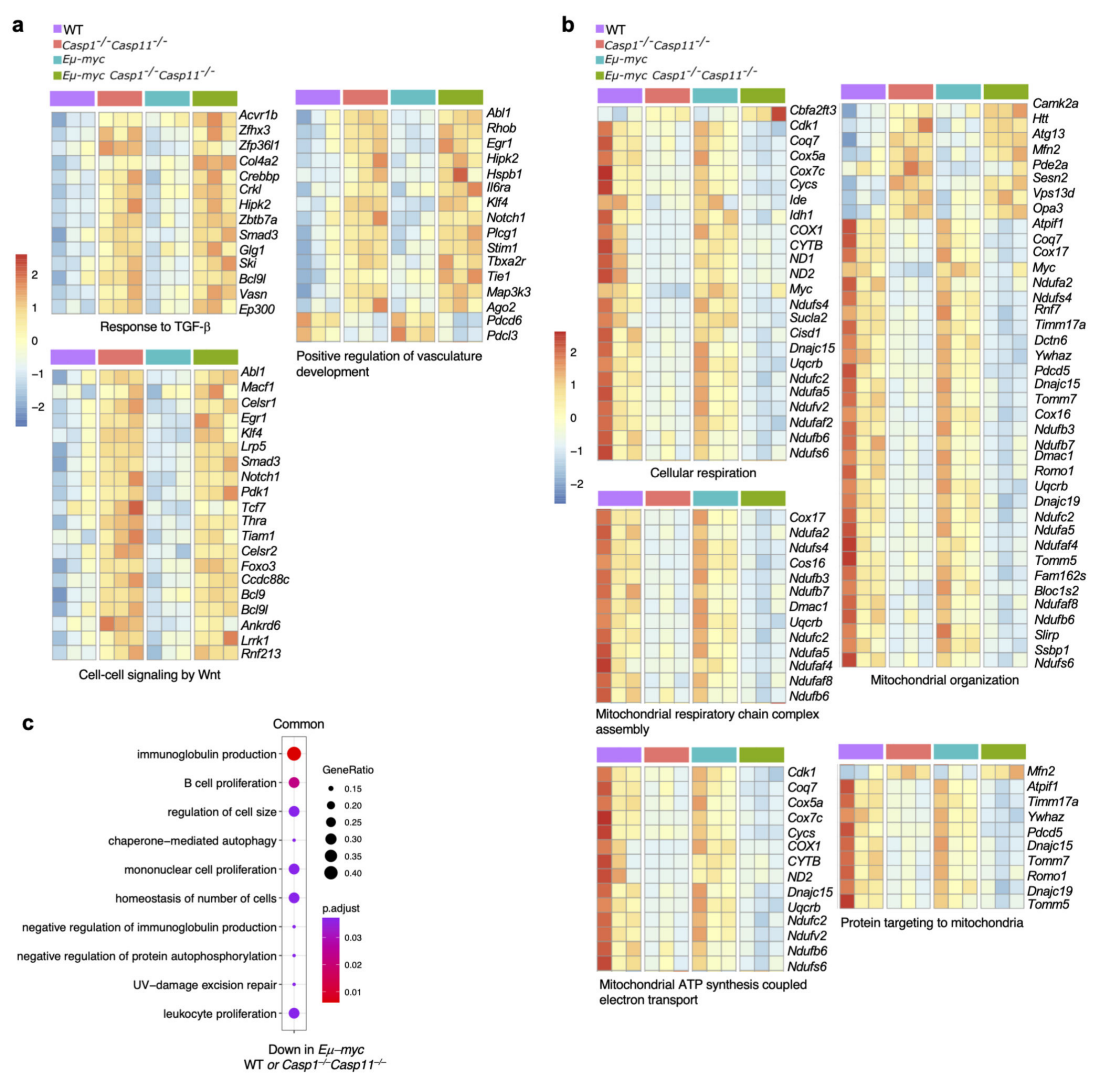


GO enrichment analysis of *Casp1*^*−/−*^*Casp11*^*−/−*^-associated genes common to both non-transgenic and *Eμ-myc* backgrounds showed significant enrichments in numerous GO terms, with many cell cycle related GO term genes expressed at higher levels (upregulated) in LSK HSCs from *Casp1/Casp11*-deficient versus *Casp1/Casp11*-sufficient mice irrespective of *Eμ-myc* genotype (Fig. 4d). Among differentially upregulated genes with the highest statistical significance were genes involved in Ras protein signal transduction, but also response to TGF-β and cell-cell signaling by Wnt (Fig. 4d and Supplementary Data 1). Several cell proliferation-related GO pathways also achieved high statistical significance and were comprised of genes shared with Ras signaling, Wnt signaling, and response to TGF-β, but also *Cdk6* (cyclin-dependent kinase 6), *Mpl* (myeloproliferative leukemia virus oncogene), *Numa1* (nuclear mitotic apparatus protein), *Rptor* (regulatory associated protein of MTOR), and others (Extended Data Fig. 5a and Supplementary Data 2). Common *Casp1*^*−/−*^*Casp11*^*−/−*^-associated genes expressed at lower levels (downregulated) in LSK HSCs showed GO term enrichment in mitochondria related processes (Fig. 4d and Extended Data Fig. 5b), which reflects potentially suppressed mitochondrial function in rapidly proliferating *Casp1/Casp11*-deficient HSCs^32,33^. Fewer GO enrichments were shown in the *Eμ-myc*-associated genes common to both *Casp1/Casp11*-deficient and sufficient backgrounds (Extended Data Fig. 5c). Collectively, these data revealed an HSC transcriptome characterized most significantly by Ras signaling and genes involved in cell cycling concordant with the increased HSC proliferation we had noted in *Eμ-myc Casp1*^*−/−*^*Casp11*^*−/−*^ and *Casp1*^*−/−*^*Casp11*^*−/−*^ mice.

## The stromal inflammasome suppresses HSC Ras signaling

We next assessed protein and transcript expression of a select number of Ras pathway related genes in LSK HSCs from WT, *Eμ-myc, Casp1*^*−/−*^*Casp11*^*−/−*^, and *Eμ-myc Casp1*^*−/−*^*Casp11*^*−/−*^ mice. By flow cytometry, we confirmed that Ras signal transduction associated surface platelet derived growth factor receptor beta (PDGFRb)^34^ and Notch-1^35,36^ were highly expressed on a bulk LSK HSC and per cell basis in premalignant *Casp1*^*−/−*^*Casp11*^*−/−*^ mice compared to WT controls, and the effect was further magnified in *Eμ-myc Casp1*^*−/−*^*Casp11*^*−/−*^ mice (Fig. 4f,g). This increased protein expression mirrored the increased transcript expression levels of Ras pathway related (Kinase suppressor of Ras1 *Ksr1*, Synaptic Ras GTPase-activating protein 1 *Syngap1, Abl1* proto-oncogene, Ras protein activator like 1 *Rasal1*, and T cell lymphoma invasion and metastasis 1 *Tiam1*) and also cancer related genes (*Pdgfrb, Notch1, Cbl* proto-oncogene, *Trio* and Ras homology family member b *Rhob*) in *Casp1*^*−/−*^*Casp11*^*−/−*^ and *Eμ-myc Casp1*^*−/−*^*Casp11*^*−/−*^ LSK HSCs (Fig. 4h). Together, these data supported increased Ras pathway-associated molecular representation upon inflammasome impairment.

**Figure 5:**
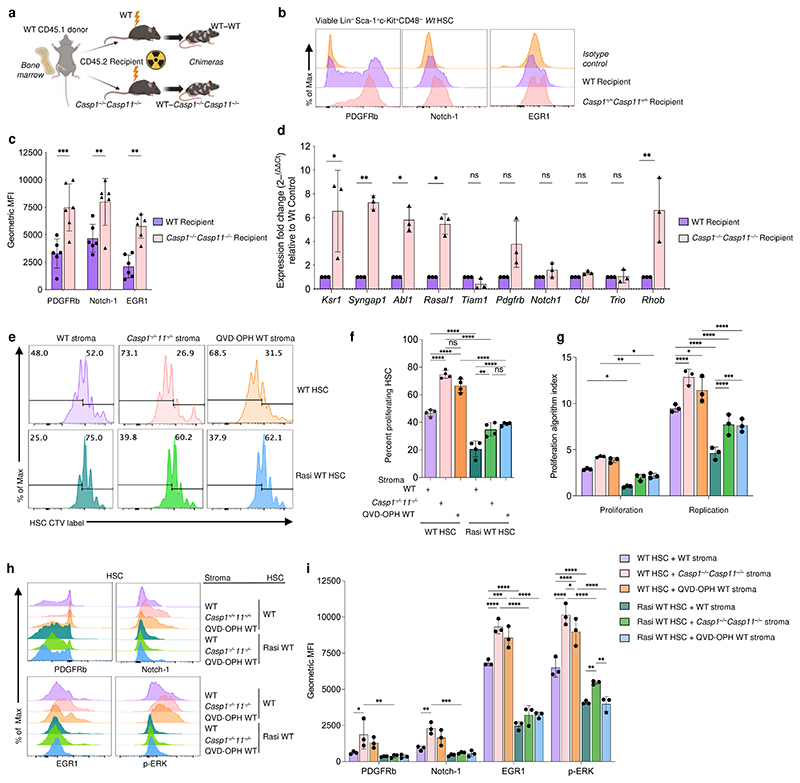
The stromal inflammasome constrains the Ras pathway in HSCs. **a**, Schematic of bone marrow chimeric mouse experimental design to investigate the role of Caspase-1/11 in bone marrow stroma on wild type bone marrow derived HSC phenotype and gene expression. Created with BioRender.com and exported under a paid subscription to BioRender. **b**, Representative histograms of HSC (Lin^–^Sca1^+^c-Kit^+^CD48^–^) Ras normalized target expression of wild type WT donor CD45.1^+^ CD45.2^–^ bone marrow from chimeric WT CD45.2^+^ recipient vs *Casp1*^*−/−*^*Casp11*^*−/−*^ CD45.2^+^ recipient. **c**, Quantification of gMFI of (**b**); n=6 biological replicates per genotype, p**≤0.01, p***≤0.001; 2 WAY Anova. **d**, Quantitative real time PCR showing the fold change expression of Ras signaling associated, and cancer related genes derived from RNA-Seq data relative to WT (normalized to β2M at n=1) of enriched wild type donor CD45.1^+^ CD45.2^–^ bone marrow HSC from chimeric WT CD45.2 recipient vs *Casp1*^*−/−*^*Casp11*^*−/−*^ CD45.2^+^ recipient; n=3 biological replicates per genotype; p**£0.01, p***£0.001; ordinary 2 WAY Anova with Sidak’s multiple comparison posttest comparing the mean of each genotype row. **e**, Representative histograms of cell trace violet (CTV) proliferation dye labeled HSCs (CD45^+^ Sca1^+^ c-Kit^+^ CD48^–^) at 8 h timepoint from coculture of WT HSC or 4 h Ras inhibitor treated WT HSC with enriched stroma (CD45^–^Sca-1^–^c-Kit^–^Lin^–^) from WT, *Casp1*^*−/−*^*Casp11*^*−/−*^ mice or WT stroma treated with pan caspase inhibitor QVD-OPH for 2 h at 1:2 ratio (HSC:Stroma). **f**, Quantification of (**e**) as the frequency (%) of total HSCs that have undergone at least 4 rounds of proliferation (CTV^+^ left gate = proliferation dye dilution); n=4 biological replicates per condition, p**≤0.01, p****≤0.0001; 2 WAY Anova. **g**, Quantification of the proliferation and replication (expansion) index from (**e**); n=3 biological replicates per condition, p*≤0.05, p**≤0.01, p****≤0.0001; 2 WAY Anova. **h**, Representative histograms of enriched HSC (CD45^+^Sca1^+^c-Kit^+^CD48^–^) Ras normalized target expression from 16 h coculture of HSC and stroma as described in (**e**). **i**, Quantification of gMFI of (**h**); n=3 biological replicates per condition, p*≤0.05, p**≤0.01, p***≤0.001, p****≤0.0001; ordinary 2 WAY Anova with Tukey’s multiple comparisons posttest comparing simple effects within condition rows. For **f**,**g**, only p values ≤0.05 are shown. All data are presented as mean values +/- SD.

Next, we reconstituted lethally irradiated WT and *Casp1*^*−/−*^*Casp11*^*−/−*^ recipient mice with bone marrow from WT mice to interrogate WT HSCs that were subjected to either a WT or a caspase-1/11 deficient bone marrow microenvironment *in vivo* (Fig. 5a). We found a significant increase in Ras signaling pathway related protein and gene transcript levels in WT HSCs from *Casp1*^*−/−*^*Casp11*^*−/−*^ compared to WT recipients, including PDGFRb, Notch-1, and EGR1 (Fig. 5b-d). Using an *ex vivo* approach, we cocultured isolated WT HSCs with either a WT, *Casp1*^*−/−*^*Casp11*^*−/−*^, or WT stroma treated with the pan-caspase inhibitor QVD-OPH. Concordant with the increased stem and progenitor cell proliferation we had noted in *Casp1*^*−/−*^*Casp11*^*−/−*^ mice, WT HSCs proliferated and expanded significantly more when cocultured with either *Casp1*^*−/−*^*Casp11*^*−/−*^ or QVD-OPH-treated WT stroma compared to untreated WT stroma (Fig. 5e *top row* and quantified in Fig. 5f,g). Coculture of WT HSCs with *Casp1*^*−/−*^*Casp11*^*−/−*^ stroma also significantly increased HSC expression of PDGFRb, Notch1, EGR1, and phosphorylated extracellular signal-regulated kinase (p-ERK), directly downstream of Ras signaling^37^, compared to HSCs cocultured with WT stroma (Fig. 5h and quantified in Fig. 5i). Supporting caspase-dependency, treatment of WT stroma with QVD-OPH phenocopied the *Casp1*^*−/−*^*Casp11*^*−/−*^ stroma showing significantly increased proliferation (Fig. 5e *top row* and 5f, g), as well as increased PDGFRb, Notch-1, EGR1, and phospho-ERK levels in cocultured HSCs as compared to HSCs cocultured with untreated WT stroma (Fig. 5h and quantified in Fig. 5i).

Lastly, WT HSCs treated with the Ras inhibitor BI-2852 were refractory to the effects of the *Casp1*^*−/−*^*Casp11*^*−/−*^ stroma whereby surface levels of PDGFRb, Notch-1, EGR1, and phospho-ERK were all significantly decreased compared to those in WT HSCs on *Casp1*^*−/−*^*Casp11*^*−/−*^ stroma (Fig. 5h and quantified in Fig. 5i). The expression of EGR1 and p-ERK was particularly sensitive to Ras inhibition even in WT HSCs, confirming its association with Ras signaling (Fig. 5h and quantified in Fig. 5i). Ras inhibition also significantly decreased HSC proliferation upon coculture with either WT or *Casp1*^*−/−*^*Casp11*^*−/−*^ stroma (Fig. 4e *bottom row* and quantified in Fig. 4f,g). Collectively, these results supported findings from the transcriptional HSC profiling and highlighted our conclusion that inflammasome activity within the stroma inhibits Ras signal transduction in HSCs.

## Stromal inflammasome control of HSC TNF and MIP receptors

**Extended Data Figure 6:**
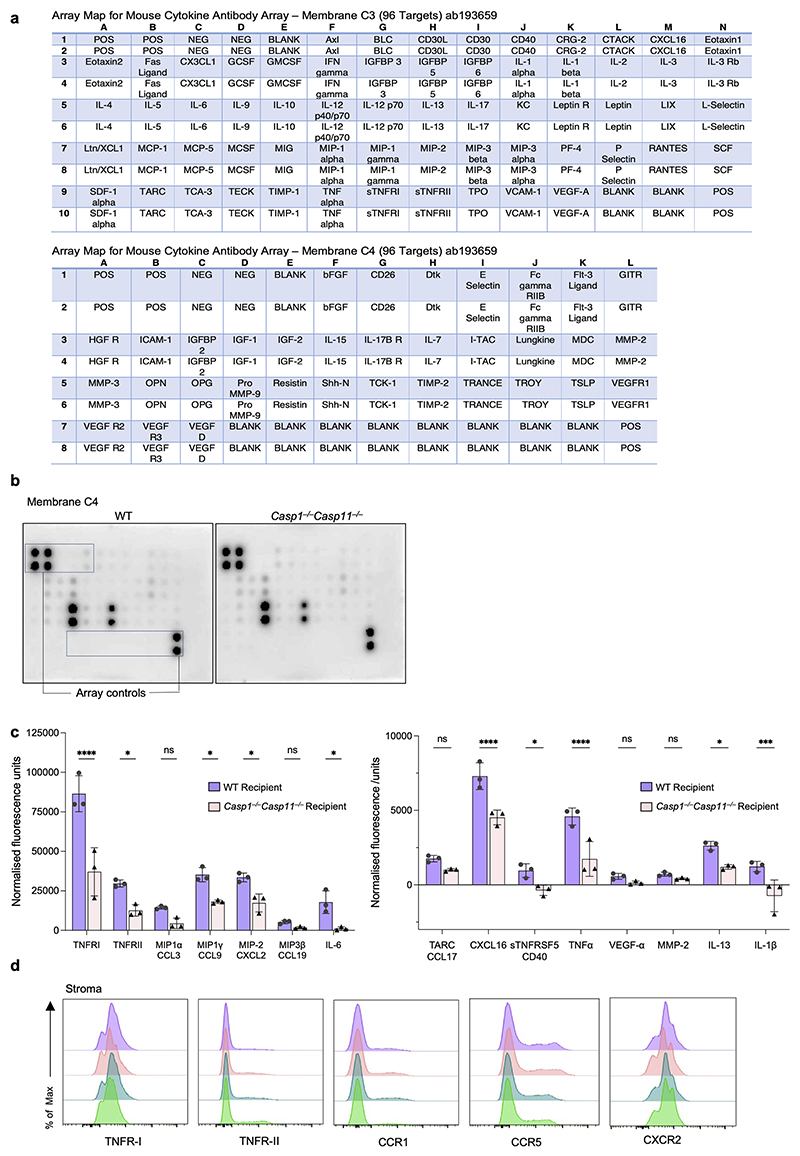


**Figure 6:**
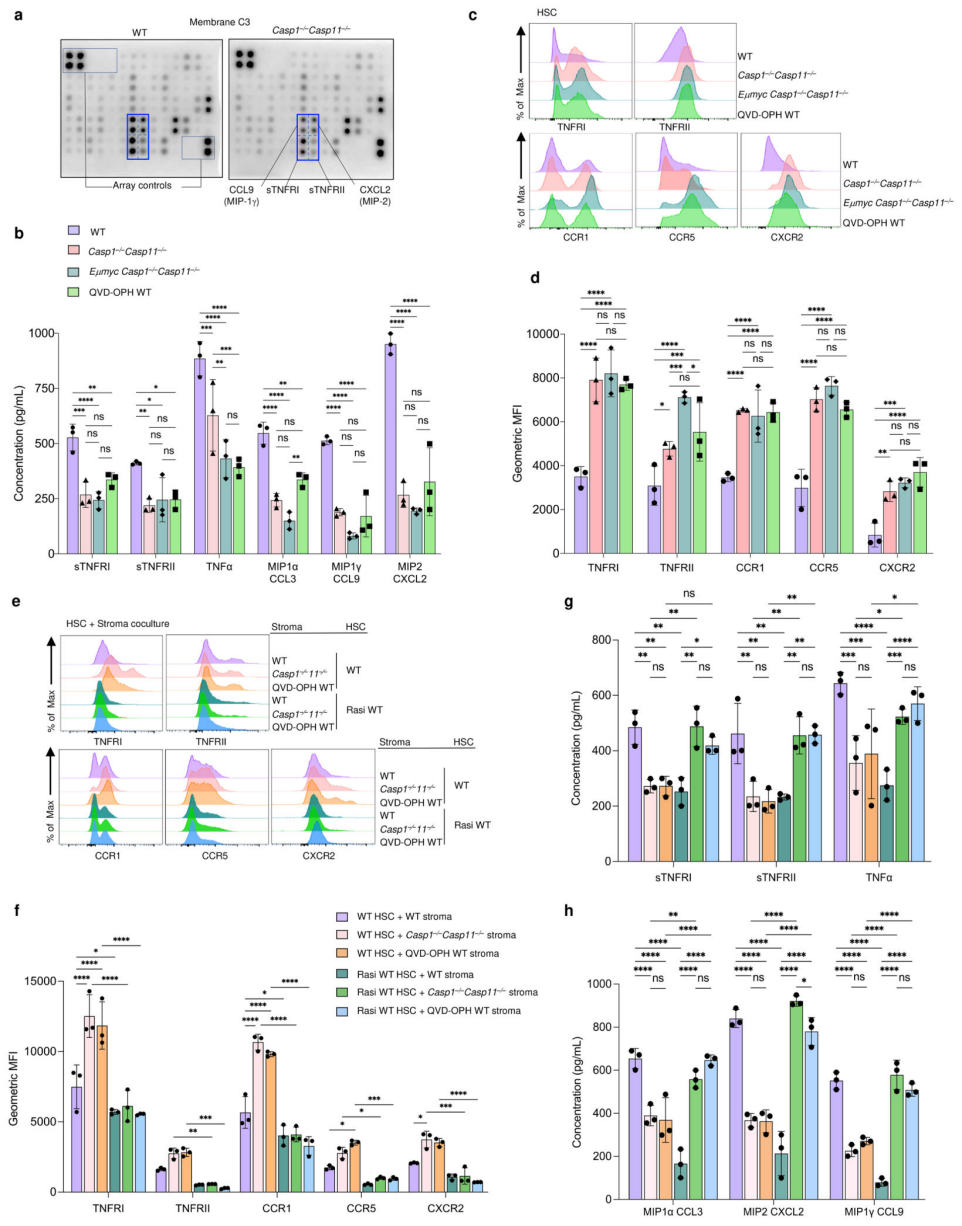
The stromal inflammasome controls HSC surface TNFR1/II and MIP receptors via Ras. **a**, Representative cytokine array blots of WT (left) vs *Casp1*^*−/−*^*Casp11*^*−/−*^ (right) bulk bone marrow after 6 h incubation including array controls of kit Membrane C3. **b**, Concentration of soluble sTNFR1 and sTNFRII, TNF and indicated chemokines measured by ELISA at 6 h post *ex vivo* culture of bone marrow from adult male WT, *Casp1*^*−/−*^*Casp11*^*−/−*^, and *Eμ-myc Casp1*^*−/−*^*Casp11*^*−/−*^ mice or WT bone marrow treated with pan caspase inhibitor QVD-OPH for 2 h. n=3 biological replicates per genotype or condition, ns, p>0.05, p*≤0.05, p**≤0.01, p***≤0.001, p****≤0.0001, 2 WAY Anova. **c**, Representative histograms of indicated receptor expression on HSC (CD45^+^Lin^–^Sca1^+^c-Kit^+^CD48^–^) from (**b**) at 6 h post *ex vivo* culture. **d**, Quantification of gMFI of (**c**). **e**, Representative histograms of indicated receptor expression on enriched HSC (CD45^+^ Lin^–^Sca1^+^c-Kit^+^CD48^–^) at 16 h of coculture of WT HSC or 4 h Ras inhibitor treated WT HSC with enriched stroma (CD45^–^Sca-1^–^c-Kit^–^Lin^–^) from WT, *Casp1*^*−/−*^*Casp11*^*−/−*^ mice or WT stroma treated with pan caspase inhibitor QVD-OPH for 2 h at 1:2 ratio (HSC:Stroma); n=3 biological replicates per genotype or condition, ns, p>0.05, p****≤0.0001, 2 WAY Anova. **f**, Quantification of gMFI of (**e**); n=3 biological replicates per genotype or condition, p*≤0.05, p****≤0.0001, ordinary 2 WAY Anova with Tukey’s multiple comparisons posttest comparing simple genotype column effects within rows. **g**, Concentration of sTNFR1, sTNFRII, and TNF from (**e**) cocultures as measured by ELISA; n=3 biological replicates per genotype or condition, ns, p >0.05, p*≤0.05, p**≤0.01, p****≤0.0001, 2 WAY Anova. **h**, Concentration of indicated MIP family chemokines from (**e**) cocultures as measured by ELISA; n=3 biological replicates per genotype or condition, ns, p>0.05, p****≤0.0001, 2 WAY Anova. **b**,**d**, Both share the same legend shown in **b. g**,**f**,**h**, All share the same legend shown in **f**. For **f**, only p values ≤0.05 are shown. All data are presented as mean values +/- SD.

To further probe the properties of *Casp1*^*−/−*^*Casp11*^*−/−*^ stroma, we interrogated the whole bone marrow from premalignant mice with a mouse cytokine antibody array. Of 96 potential targets, the most abundant signals belonged to soluble (s)TNFRI (TNFRSF1A), sTNFRII (TNFRSF1B), and macrophage inflammatory protein (MIP) family members CCL9 (MIP-1γ) and CXCL2 (MIP-2), and these were significantly lower in *Casp1*^*−/−*^*Casp11*^*−/−*^ bone marrow (Fig. 6a and Extended Data Fig. 6a-c). Other less-abundant signals belonging to TNF, IL-6, CXCL16, CD40, IL-13, similar to IL-1β and IL-18, were also significantly reduced in the *Casp1*^*−/−*^*Casp11*^*−/−*^ bone marrow (Fig. 6a and Extended Data Fig. 6a-c). We measured significantly lower concentrations of sTNFRI, sTNFRII, TNF-α, CCL9, CXCL2, but also CCL3 (MIP1α) *ex vivo* in bulk bone marrow from premalignant *Casp1*^*−/−*^*Casp11*^*−/−*^ and similarly *Eμ-myc Casp1*^*−/−*^*Casp11*^*−/−*^ mice compared to WT mice (Fig. 6b). Treatment of WT bone marrow with QVD-OPH *ex vivo* phenocopied the results in caspase-1/11 deficient mice (Fig. 6b). Examining the levels of the corresponding surface receptors on HSCs, we found significantly higher levels of TNFRI, TNFRII, and receptors for CCL3 and CCL9 (CCR1), CCL3 (CCR5) and CXCL2 (CXCR2) on *Casp1*^*−/−*^*Casp11*^*−/−*^, *Eμ-myc Casp1*^*−/−*^*Casp11*^*−/−*^, or QVD-OPH-treated bone marrow compared to WT bone marrow HSCs (Fig. 6c,d). QVD-OPH-treatment of the bone marrow *ex vivo* negated the significant differences in expression of most of these receptors on HSCs from WT compared to *Casp1*^*−/−*^*Casp11*^*−/−*^ or *Eμ-myc Casp1*^*−/−*^*Casp11*^*−/−*^ bone marrow (Fig. 6c,d). There was no appreciable expression of TNFRII and CCR1 in the CD45^–^ bone marrow stromal component, while the expression of TNFRI, CCR5 and CXCR2 were similar under all conditions (Extended Data Fig. 6d).

Culture of WT LSK HSCs with stromal cells from either *Casp1*^*−/−*^*Casp11*^*−/−*^ or QVD-OPH treated WT stroma also significantly increased TNFRI, CCR1 and CXCR2 surface levels compared to HSC cultured with WT stroma, with more modest increases in TNFRII and CCR5 (Fig. 6e,f). Levels of the soluble TNF receptors (sTNFRI and sTNRFII), TNF and MIP ligands were lower in cocultures of WT HSC with inflammasome-deficient *Casp1*^*−/−*^*Casp11*^*−/−*^ or QVD-OPH-treated WT stroma (Fig. 6g).

**Extended Data Figure 7:**
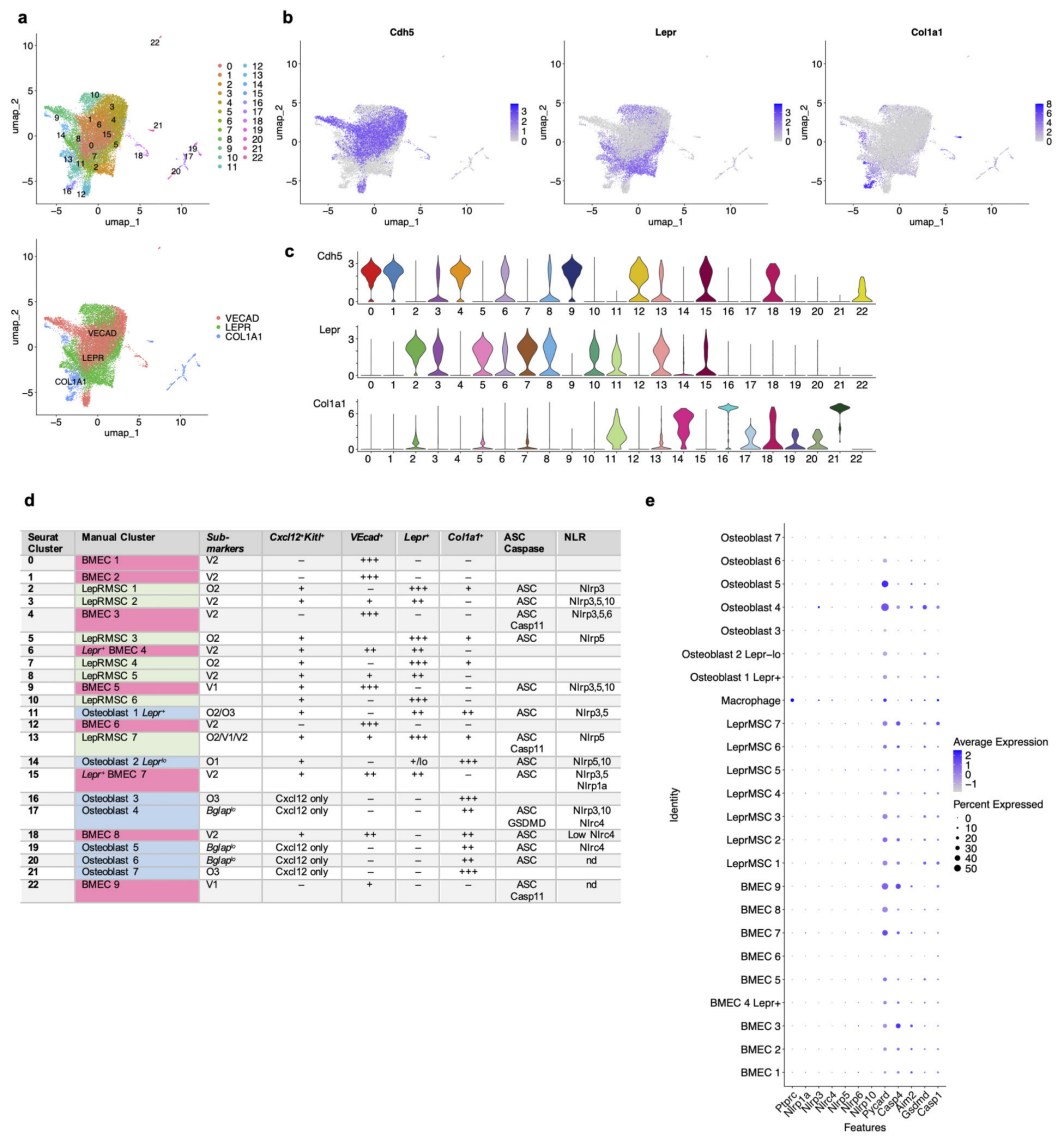


Lastly, to test whether the increased HSC, TNF and MIP receptor levels we noted reflected increased Ras signaling in HSCs upon coculture with caspase-1/11 deficient stroma, we treated WT HSC with Ras inhibitor BI-2852 and cocultured them with *Casp1*^*−/−*^*Casp11*^*−/−*^ or QVD-OPH treated WT stroma compared to untreated WT stroma as controls. Ras inhibition made HSCs refractory to the effects of the caspase-1/11 deficient stroma and significantly prevented the caspase 1/11 deficient stroma-driven increase in TNFRI/II and MIP receptor levels. This effect ranged from 50% reduction in TNFRI and CCR1 levels and even greater for TNFRII, CCR5 and CXCR2 compared to <10% reduction in levels in the presence of WT stroma (Fig. 6e,f). Concordantly, Ras inhibitor BI-2852 treatment of HSCs restored the levels of soluble TNFR and MIP ligands back to WT levels only in the *Casp1*^*−/−*^*Casp11*^*−/−*^ or QVD-OPH treated WT stroma and not untreated WT stroma cocultures (Fig. 6g). Collectively, these results show that the stromal inflammasome controls the levels of TNF and MIP receptors on HSCs by restraining Ras signaling (Extended Data Fig. 7).

## A stromal NLRP3 inflammasome controls HSC Ras signaling

**Extended Data Figure 8:**
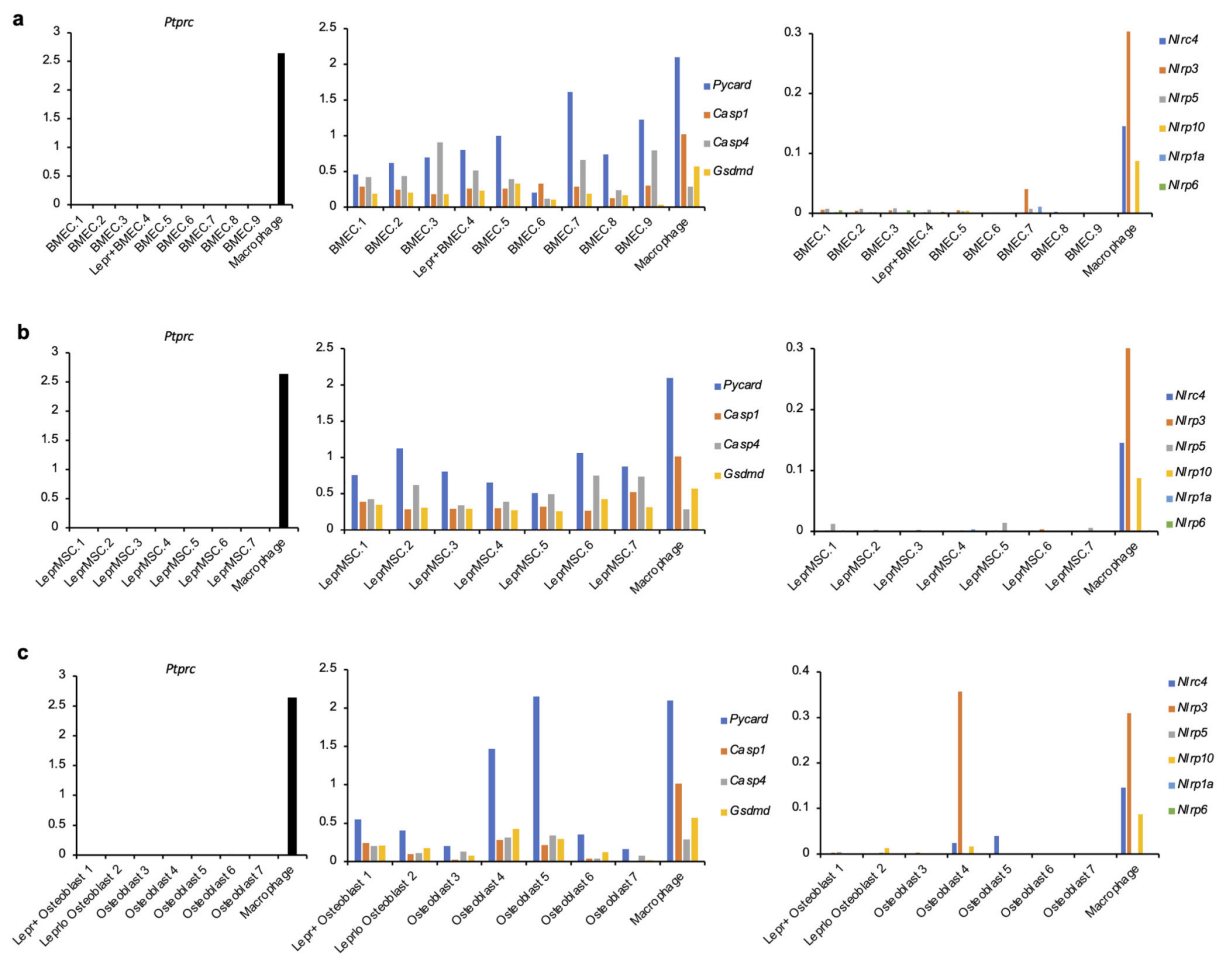


We next mined single cell RNA-sequencing of bone marrow stromal cells for the expression of inflammasome related genes. We chose a dataset on the major stromal populations shown to support HSC^38^, including leptin receptor expressing (*Lepr*^+^) mesenchymal stromal cells (MSC), vascular endothelial cells marked by *Cdh5* (VE-cadherin) expression^39,40^, and osteoblasts marked by *Col1a1* (collagen type 1, alpha 1) expression^41,42^. Uniform manifold Approximation and Projection (UMAP) displayed 23 distinct clusters among these stromal cells and a distinct distribution pattern of niche markers (*Cdh5, Lepr* and *Col1a1*) (Extended Data Fig. 7a,b). Visualization of the frequency of niche marker genes across the 23 clusters led to a first-tier cluster annotation into either bone marrow endothelial cells (BMEC), LEPR^+^ MSC, or osteo-lineage cells (Extended Data Fig. 7c), which were subjected to a second-tier manual annotation based on the combined expression of sub-markers associated with specialized subsets within each major HSC-niche stromal cell type^38^. This iteration revealed nine BMEC, seven LepR^+^ MSC, and seven osteoblast clusters with different sub-marker distributions (Extended Data Fig. 7d). Of inflammasome-associated genes, we detected varying expression levels of *Casp1* and *Casp11* (*Casp4*), but also *Pycard* (encoding the inflammasome adaptor protein ASC^2^), and *Gsdmd*^43^ (Extended Data Fig. 7e). The stromal expression levels of these genes were in some cases comparable to those in bone marrow macrophages (Extended Data Fig. 8). Sensor NLR levels were comparatively lower than those in macrophages with the exception of an osteoblast cluster which expressed *Nlrp3* at levels similar to those in macrophages (Extended Data Fig. 8).

**Extended Data Figure 9:**
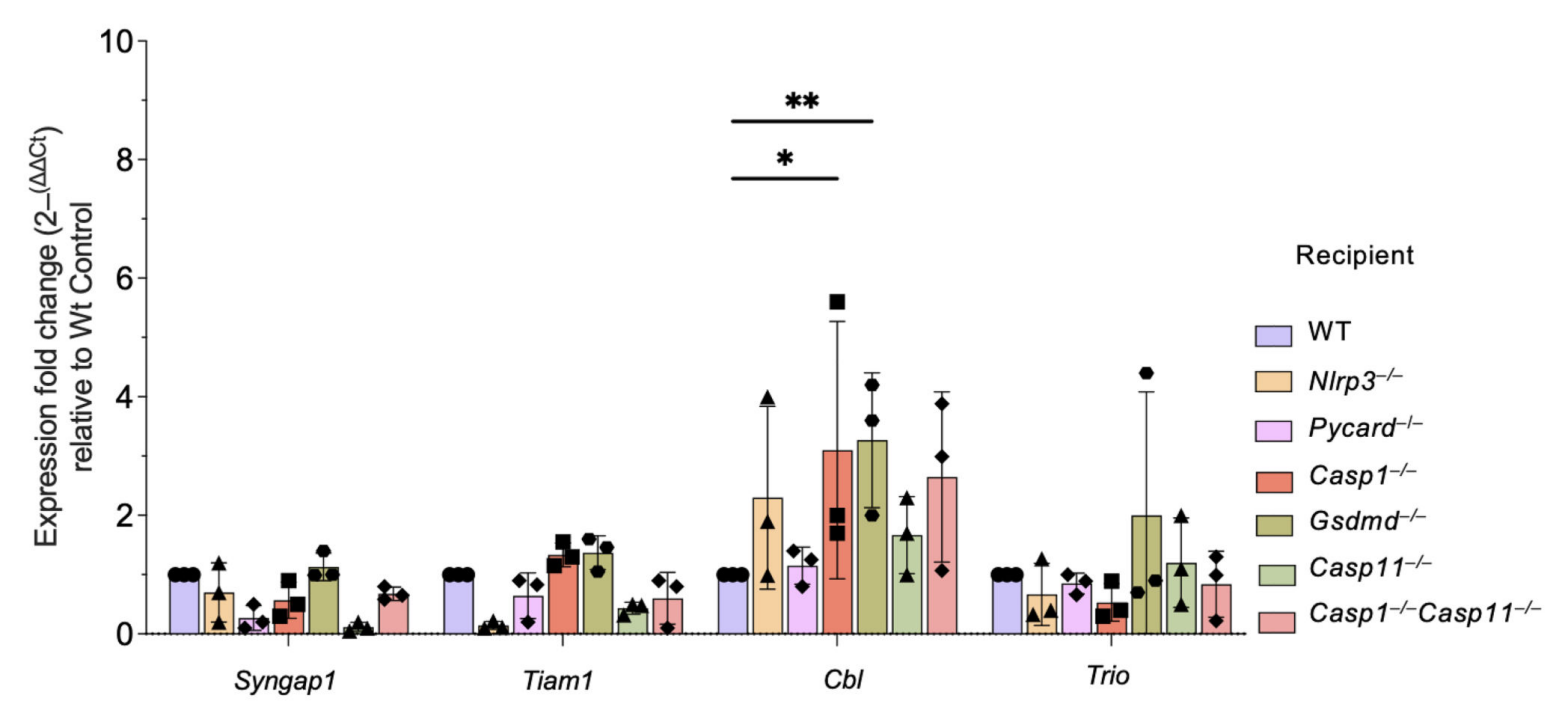


**Figure 7:**
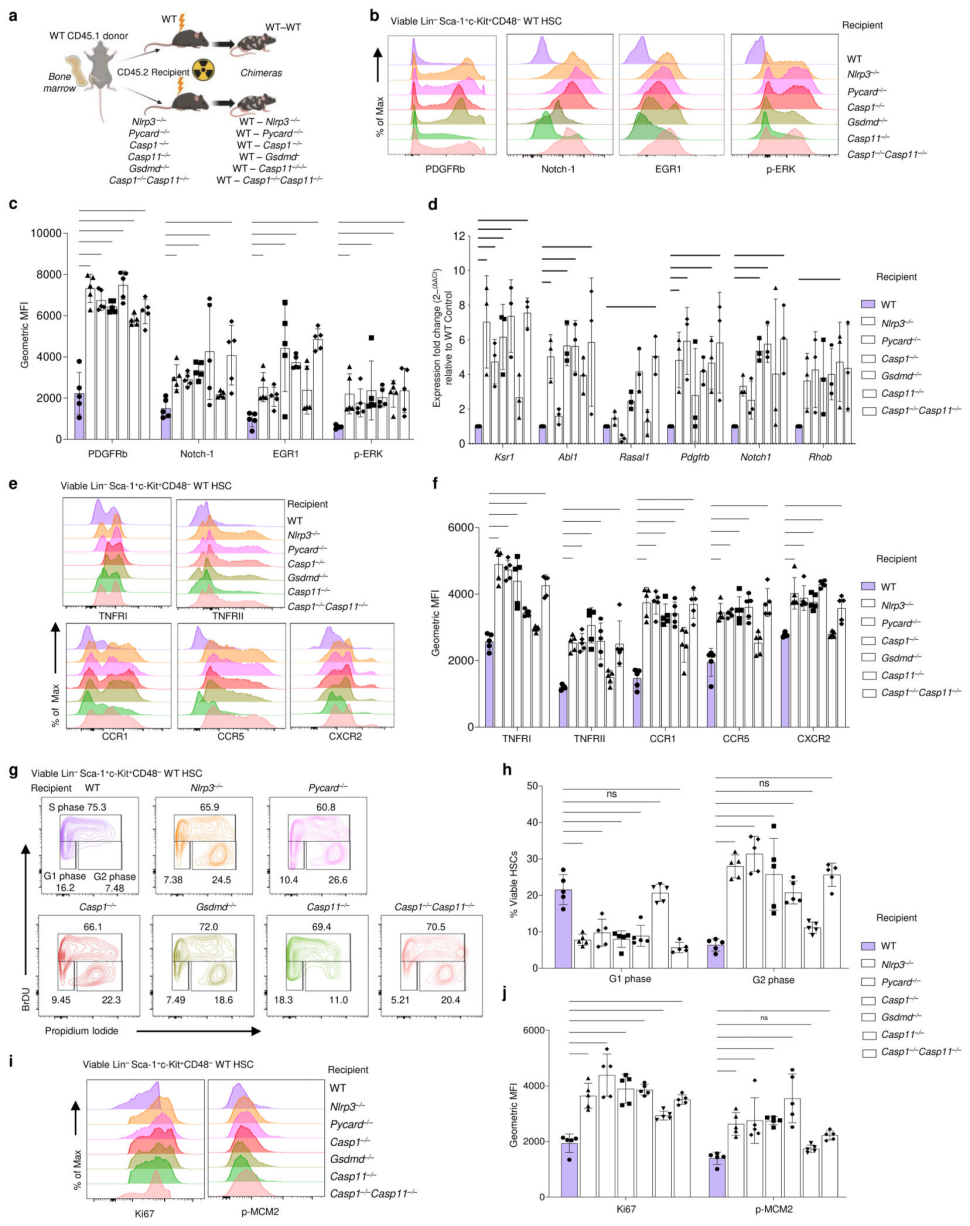
Canonical NLRP3 inflammasome components control HSC Ras signaling in trans. **a**, Schematic of bone marrow chimeric mouse experimental design to investigate the role of various bone marrow stromal inflammasome components as indicated on wild type bone marrow derived HSC phenotype and gene expression. **b**, Representative histograms of HSC (Lin^–^Sca1^+^c-Kit^+^CD48^–^) Ras normalized target expression of WT donor CD45.1^+^ CD45.2^–^ bone marrow from chimeric WT recipient vs *knockout* recipient. **c**, Quantification of gMFI of (**b**); n=5 biological replicates per genotype recipient, p*≤0.05, p***≤0.001, p****≤0.0001, 2 WAY Anova. **d**, Quantitative real time PCR showing the fold change expression of Ras signaling associated, and cancer related genes derived from RNA-Seq data relative to WT (normalized to β2M at n=1) of enriched wild type donor CD45.1^+^ CD45.2^–^ bone marrow from chimeric WT recipient vs *knockout* recipients; n=3 biological replicates per genotype recipient, p*<0.05, p**≤0.01, p****≤0.0001, ordinary 2 WAY Anova with Dunnett’s multiple comparisons posttest posttest comparing simple genotype column effects within rows. **e**, Representative histograms of TNF receptor and MIP receptor expression on HSCs from WT donor CD45.1^+^ CD45.2^–^ bone marrow from chimeric WT recipient vs *knockout* recipient. **f**, Quantification of gMFI of (**e**); n=5 biological replicates per genotype recipient, p**≤0.01, p****≤0.0001, 2 WAY Anova. **g**, Representative contour plots of proliferation marker BrDU and DNA synthesis marker PI on HSCs from WT donor CD45.1^+^ CD45.2^–^ bone marrow from chimeric WT recipient vs *knockout* recipient. **h**, Quantification of the frequency of viable HSCs from (**g)**; n=5 biological replicates per genotype recipient, ns, p>0.05, p****≤0.0001, ordinary 2 WAY Anova with Sidak’s multiple comparisons posttest comparing simple genotype column effects within rows. **i**. Representative histograms of HSC (Lin^–^Sca1^+^c-Kit^+^CD48^–^) proliferation marker expression of WT donor CD45.1^+^ CD45.2^–^ bone marrow from chimeric WT recipient vs *knockout* recipient. **j**, Quantification of gMFI of (**i**); n=5 biological replicates per genotype recipient, ns, p>0.05, p**≤0.01, p****≤0.0001, 2 WAY Anova. For **c, d, f**, only p values ≤0.05 are shown. All data are presented as mean values +/-SD.

Prompted by these findings, we reconstituted lethally irradiated CD45.2 mice deficient in specific components of the NLRP3 inflammasome (*Nlrp3*^*−/−*^, *Pycard*^*−/−*^, *Gsdmd*^*−/−*^) and singly deficient in either *Casp1*^*−/−*^ or *Casp11*^*−/−*^ with bone marrow from CD45.1 WT mice (Fig. 7a). We compared our various readouts of HSC Ras signaling in these mice to WT or *Casp1*^*−/−*^*Casp11*^*−/−*^ recipient chimeric mice as a reference (Fig. 7a). We found that all Ras signaling pathway related protein and gene transcript levels were significantly increased in WT LSK HSCs from recipient *Nlrp3*^*−/−*^, *Pycard*^*−/−*^, *Casp1*^*−/−*^ and *Gsdmd*^*−/−*^ chimeric mice, including HSC surface protein levels of PDGFRb, Notch-1, and EGR1 (Fig. 7b-d and Extended Data Fig. 9). The stromal effects were marginal to not significantly different in *Casp11*^*−/−*^ compared to WT chimeric mice for Notch-1, EGR1 and a number of Ras target genes including *Ksr1, Syngap1, Rasal1, Tiam1, Cbl* and *Trio* (Fig. 7b-d). WT HSCs isolated from recipient *Nlrp3*^*−/−*^, *Pycard*^*−/−*^, *Casp1*^*−/−*^ or *Gsdmd*^*−/−*^ chimeric mice also showed significantly increased TNFRI, TNFRII, CCR1, CCR5 and CXCR2 compared to those from WT chimeric mice (Fig. 7e,f). *Casp1*^*−/−*^ and not *Casp11*^*−/−*^ stroma most closely phenocopied the effects of *Casp1*^*−/−*^*Casp11*^*−/−*^ stroma on WT HSC expression of TNF and MIP receptors (Fig. 7d,f).

Concordant with the increased stem and progenitor cell proliferation we had noted in *Casp1*^*−/−*^*Casp11*^*−/−*^ mice, a significantly higher proportion of WT HSCs entered G2 phase of cell cycle when isolated from chimeric mice bearing an NLRP3, ASC, *Casp1* or GSDMD deficient stroma compared to WT stroma-bearing chimeric mice (Fig. 7g,h). HSC expression of the proliferation marker Ki67 was significantly higher in each case when stroma was deficient for the tested inflammasome components, but the levels of phosphorylated mini-chromosome maintenance protein (p-MCM2), required for initiation and elongation of DNA replication, were significantly increased in all cases except for the WT HSC isolated from *Casp11*^*−/−*^ stroma bearing chimeric mice (Fig. 7i and quantified in Fig. 7j). Collectively, these results support the conclusion that a canonical NLRP3 inflammasome in the stroma played a key role in inhibiting HSC Ras signal transduction, controlling HSC surface levels of TNF and MIP receptors, and restraining HSC replication.

## Soluble and cell contact inflammasome control of HSCs

**Figure 8:**
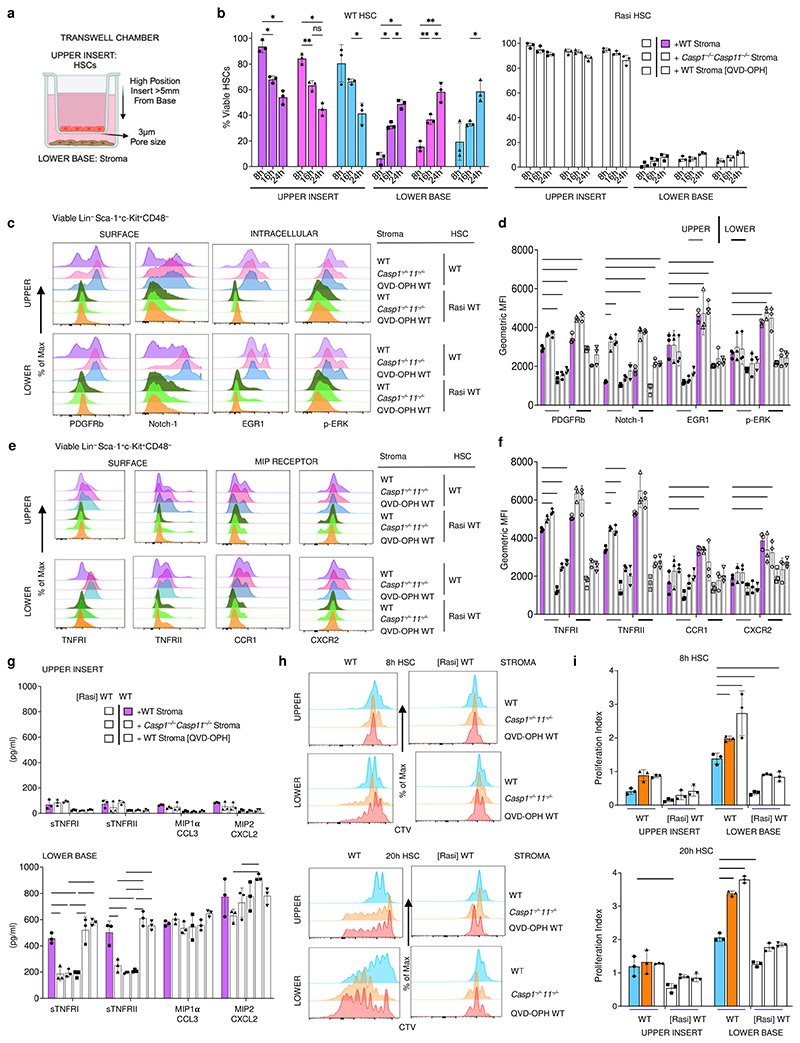
Stromal-derived soluble factor and direct stroma-HSC contact mediate stromal inflammasome control of HSCs. **a**. Schematic of experimental transwell setup showing HSC in the top insert separated from bone marrow stroma in the lower base by a 3μM pore size allowing exchange of soluble factors and HSC migration towards the stroma. **b**. Quantification of viable Sca1^+^ cKit^+^ cells with (right) Ras inhibitor treatment and untreated (left) of total CD45^+^ cells in the upper insert and lower base every 8hrs during coculture with WT stroma, *Casp1*^*−/−*^*Casp11*^*−/−*^ stroma or WT stroma treated with pan caspase inhibitor QVD-OPH, n=3 biological replicates per condition, ns, p>0.05, p*≤0.05, p**≤0.01, 2 WAY Anova. **c**, Representative histograms of WT HSC (Lin^–^Sca1^+^c-Kit^+^CD48^–^) Ras normalized target expression during coculture with WT stroma, *Casp1*^*−/−*^*Casp11*^*−/−*^ stroma or WT QVD-OPH treated stroma. **d**, Quantification of gMFI of (**c**), n=3 biological replicates per condition, p*≤0.05, p**≤0.01, p****≤0.0001, 2 WAY Anova. **e**, Representative histograms of TNF and MIP receptor expression on HSCs during coculture with WT stroma, *Casp1*^*−/−*^*Casp11*^*−/−*^ stroma or WT QVD-OPH treated stroma. **f**, Quantification of gMFI of (**e**), n=3 biological replicates per condition, p*≤0.05, p****≤0.0001, 2 WAY Anova. **g**, Quantification of cytokines - soluble TNFR and MIP family in the upper HSC (top) and lower stroma (bottom) portion of transwell, n=3 biological replicates per condition, p**≤0.01, p***≤0.001, p****≤0.0001, 2 WAY Anova. **h**, Representative histograms of TNF and MIP receptor expression on WT and Ras inhibitor treated HSCs during coculture with WT stroma, *Casp1*^*−/−*^*Casp11*^*−/−*^ stroma or WT QVD-OPH treated stroma at 8hr (top) and 20hr (bottom). **i**, Quantification of proliferation index of (**h**), n=3 biological replicates per condition, p*≤0.05, p****≤0.0001, 2 WAY Anova. For **d, f, g, i**, only p values ≤0.05 are shown. All data are presented as mean values +/- SD.

To understand whether the trans-acting effect of the stromal inflammasome on HSCs was dependent on soluble or cell contact-dependent factors, we conducted a series of transwell migration assays where we separated HSCs in upper inserts from the bone marrow stroma in lower chambers of transwells (Fig. 8a). Top insert HSCs migrated towards the stroma lower base as soon as 8 hours post-transwell setup, and to similar levels irrespective of whether the stroma was inflammasome sufficient or deficient (Fig. 8b, left). This migration was severely abrogated upon Ras-inhibitor treatment of viable HSCs (Fig. 8b, right). Surface Ras related targets PDGFRb and Notch-1 were significantly upregulated on both top insert separated HSCs and lower base HSCs in contact with *Casp1*^*−/−*^*Casp11*^*−/−*^ and WT QVD-OPH-treated stroma compared to WT stroma, consistent with a soluble factor effect that peaks at HSC-stroma proximity (Fig. 8c,d). TNF receptors were increased on top insert and lower base HSCs with *Casp1*^*−/−*^*Casp11*^*−/−*^ and WT QVD-OPH-treated stroma compared to WT stroma, and most significantly in upper insert HSCs (Fig. 8e,f). Increases in both Ras target and TNF-receptor expression levels were abrogated upon Ras inhibitor treatment of HSCs (Fig. 8c–f). There was an accumulation of soluble TNFRI/II and MIP ligands in the lower base containing the stroma at 4 hours (Fig. 8g). The levels of surface TNFRI/II on HSCs inversely correlated with those of their respective soluble forms (Fig. 8g). On the other hand, MIP ligand levels showed no significant differences between transwells containing inflammasome sufficient WT or deficient *Casp1*^*−/−*^*Casp11*^*−/−*^/QVD-OPH treated stroma (Fig. 8g). Along with these observations, HSCs proliferated most significantly when in direct contact with *Casp1*^*−/−*^*Casp11*^*−/−*^ and WT QVD-OPH treated stroma in the lower base compared to WT stroma, and this proliferation was Ras-dependent, increased over time, and was higher than no stromal contact in upper inserts (Fig. 8h,i). Collectively, these results largely recapitulated our HSC-stroma cocultures and showed that stromal inflammasome control of the HSC Ras signaling and proliferative signature is mediated by a combination of HSC-stroma cell contact and a soluble factor.

## Discussion

The findings here indicate the existence of inflammasome-dependent innate immune suppression of lymphomagenesis during a premalignant myc-deregulated phase of disease. We show that a bone marrow stromal inflammasome comprised of components of the canonical NLRP3 inflammasome suppressed the Ras-dependent proliferative state of HSCs through soluble and contact dependent trans factors. Stromal inflammasome deficiency hastened the malignant transformation of B cells upon Myc dysregulation at an early stage of their development. A myriad of stromal cell types, including endosteal and perivascular stromal cells, regulate HSC and progenitor cell proliferation and differentiation^44,45^, and our meta-analysis on bone marrow stroma showed that stromal subtypes express inflammasome markers. While this analysis was performed at steady state, it will be important to investigate the expression of inflammasome components upon Myc deregulation and at various stages of disease pathogenesis. A future systematic approach targeting each stromal subtype will also be important to identify the key cellular players in which the inflammasome is functioning.

The choice of Ras as a target for the inflammasome is notable because overactive Ras, even in the absence of specific Ras mutations, is a central node that contributes to cellular transformation by driving uncontrolled cell proliferation, resistance to apoptosis, and evasion of growth control mechanisms^46^. Although recent analyses have placed Ras mutation rates at 19% across all cancer types in the US^47^, wild-type Ras in non-mutant-Ras cancers cooperates with oncogenic drivers upon Ras amplification or activation by gene fusion, loss of negative regulators or upregulation of positive regulators^48^. Here our data suggest inflammasome suppression of proliferative Ras signaling in HSCs constrains cellular transformation during pre-malignancy.

Targeting Ras by the inflammasome is also significant because Ras partners with Myc in one of the most common and well-documented cooperation of oncogenes in cancer^11,12,13^. Although the mechanistic basis for oncogenic collaboration between Ras and Myc remains elusive, it may involve tumor stromal modulation. Myc and Ras cooperation in lung tumorigenesis led to a highly inflamed, angiogenic and immune-suppressed tumor stroma instructed by non-hematopoietic epithelium-derived factors^11^. Inflammasome deficiency within the bone marrow stromal compartment could similarly establish a hematopoietic stem and progenitor cell state of increased Ras activity, which cooperates with Myc upon its deregulation to accelerate lymphoma onset. Indeed, concomitant expression of oncogenic Ras increases expansion and tumorigenicity of *Eμ-myc* pre-B cells, and premalignancy-to-lymphoma transition in *Eμ-myc* mice often involves spontaneous activating *Ras* mutations^14^. Our findings offer the opportunity to inform innovative therapies during the critical early stages of cancer by targeting the inflammasome to disrupt oncogenic Ras-Myc cooperation and halt or reverse progression to malignancy.

Opposite to its characteristic pro-inflammatory roles in host defense and autoinflammatory disease^2^, the constraints on Ras signaling illustrate an important homeostatic function for the inflammasome. Exertion of this function in the premalignant stages of disease is diametrically opposite to the well-established role of inflammation in promoting tumor growth^49^. The cancer-suppressive *versus* cancer-supportive paradigms bear striking similarities with the homeostatic *versus* inflammatory functions of the immune system, respectively, during normal and disease states^50^. While we focused our study on the premalignant stage in a model of B cell lymphoma, it will be important, going forward, to dissect the role of the inflammasome during premalignancy in other cancer types. Different tumor types may be differently affected depending on the nature of the driver mutations they acquire and the specific homeostatic mechanisms of their tissue of origin. Future elucidation of inflammasome function in the stroma of different tissues, and its impact on stem and progenitor cells within those tissues, should significantly advance our understanding of inflammasome-dependent regulation of tissue homeostasis in health and disease.

## Methods

### Mice

C57BL/6J, CD45.1 and *Gsdmd*^*−/−*^ mice were purchased from Jackson Laboratories. *Eμ-myc* mice were obtained from the NCI Mouse Repository^51^. *Casp1*^*−/−*^*Casp11*^*−/−*^, *Pycard*^*−/−*^ and *Nlrp3*^*−/−*^ mice were obtained from Richard Flavell, Yale University. *Eμ-myc* males were crossed to *Casp1*^*−/−*^*Casp11*^*−/−*^ females to generate *Eμ-myc Casp1*^*−/−*^*Casp11*^*−/−*^ mice. For all analyses except survival, 4-6-week-old *Eμ-myc* and *Wt* littermates and *Eμ-myc Casp1*^*−/−*^*Casp11*^*−/−*^ and *Casp1*^*−/−*^*Casp11*^*−/−*^ littermates were used. *Casp1*^*−/−*^ mice were obtained from Thirumala Kanneganti. *Casp11*^*−/−*^ mice were obtained from St. Jude’s Hospital (Thirumala Kanneganti’s laboratory) under an authorization by owner Genentech. For survival curves, cohorts of mice were monitored weekly for any signs of pathology, specifically lymphadenopathy by manual palpation. A mouse was first considered to have disease when a single palpable lymph node was identified. Mice were thereafter checked every other day for disease progression and humanely sacrificed when either their tumors surpassed 1cm^3^ or their body condition score (BCS)^52^ fell below 2. All experiments were approved by the institutional animal care and use committees of both the Icahn School of Medicine at Mount Sinai and Weill Cornell Medicine and carried out in accordance with the ‘Guide for the Care and Use of Laboratory Animals’ (NIH publication 86–23, revised 1985).

### Peripheral blood analyses

Blood samples were treated with Red Blood Cell Lysing Buffer (Sigma, R7757) prior to flow cytometry. Leukocyte counts were performed by diluting whole blood 1:20 in Turk Blood Diluting Fluid (Ricca Chemical Company, 8850-32). Blood smears were stained with Giemsa Stain (EMD, R03055-74).

### Flow Cytometry

All samples were treated with Red Blood Cell Lysing Buffer prior to staining. For intracellular cleaved caspase-3 staining, cells were fixed and permeabilized using the BD Cytofix/Cytoperm kit (BD, 554714). For *ex vivo* death kinetics, CD19^+^ cells were magnetically (MACS) isolated from whole bone marrow (BM) and incubated in RPMI 10% FBS at 37^o^C. At indicated time points, CD19^+^ cells were stained using PE Annexin V Apoptosis Detection Kit (BD, 559763) followed by flow cytometry. All FACS was performed using an LSR Fortessa cytometer (BD). FACS sorting was performed on a FACS Aria II (BD). Populations were sorted to > 95% purity. Antibodies and dyes used in FACS panels include GL-7 (B cell and T cell Antigen)-AF647 (Biolegend, Clone GL7, 1:200), CD45 BUV395 (BD Biosciences, Clone 30-F11, 1:400), CD11b-PE-Cy7 (eBioscience, Clone M1/70, 1:400), Sca-1-BV711 (BioLegend, Clone D7, 1:100), F4/80-BV421 (BioLegend, Clone BM8, 1:400), F4/80-APC (eBioscience, Clone BM8, 1:400), B220-eFluor450 (eBioscience, Clone RA3-6B2, 1:400), CD19-eFluor450 (eBioscience, Clone eBio1D3, 1:200), CD11b-APC (eBioscience, Clone M1/70, 1:400), Ly6C-Alexa700 (BioLegend, Clone HK1.4, 1:200), Ly6C-FITC (BD, AL-21, 1:200), c-kit-PECy7 (BioLegend, Clone 2B8, 1:400), MHC-II-PE (eBioscience, Clone M5/114.15.2, 1:400) CD11c-Alexa700 (eBioscience, Clone N418, 1:200), Notch1 BV421 (BioLegend, Clone HMN1-12, 1:300) [Isotype Control – Armenian Hamster IgG BV421 Clone: HTK888 (BioLegend, 1:300)], EGR1 PE (Cell Signaling Technology, Clone 44D5, 1:50) [Isotype Control – Rabbit IgG PE Clone: 02-6102. 1:100 (Thermofisher)], Pdgrfb/CD140b APC (BioLegend, Clone APB5, 1:200) [Isotype Control - Rat IgG2a,κ APC, 1:200 (BioLegend)], CD48 PerCPe710 (ThermoFisher, Clone HM28-1, 1:200), CD150 APCe780 (ThermoFisher, Clone mShad150, 1:200), p-ERK PerCP-e710 (ThermoFisher, Clone MILAN8R, 1:100), p-MCM2 APC (ThermoFisher, Clone: MCM2S139-B12, 1:100), Ki-67 Brilliant Violet 786 (ThermoFisher, Clone: SolA15, 1:50), TNFR-I FITC (ThermoFisher, Clone 55R-170, 1:100), TNFR-II APC (ThermoFisher, Clone 112, 1:200), CCR5 PE (ThermoFisher, Clone 7A4, 1:400), CXCR2 BV421 (BD Biosciences, Clone V48-2310, 1:200), CCR1 APCFire750 (Biolegend, Clone, S15040E, 1:400), Lin- FITC (Biolegend, Clone: 145-2C11, RA3-6B2, M1/70, TER-119, RB6-8C5, 1:200), CD45.1 BV510 (Biolegend, Clone: A20, 1:100), CD45 BV650 (BD Biosciences, Clone:30-F11, 1:500), CD120b primary antibody (ThermoFisher, Clone: TR75-54, 1:100), Goat Armenian Hamster IgG (+L) Highly Cross-Adsorbed secondary antibody AF594 (ThermoFisher, 1:400), CD45.2 BUV737 (BD Biosciences, Clone 104, 1:200), BrDU FITC (ThermoFisher, Clone: BU20A, 5ul/test), and Live/Dead Aqua/Blue Reactive Dye, 1:1000 (ThermoFisher) and Propidium Iodide, 1:1000 (BioLegend).

### qRT-PCR

Quantitative real time PCR was performed on sorted cell subsets using primer and FAM-BHQ-1 probe sets (BioSearch):

*Casp1* forward primer: CCGAGGGTTGGAGCTCAAG

*Casp1* reverse primer: CACCTCTTTCACCATCTCCAGA

*Casp1* probe: TGACCTCAGAGAAATGAAGTTGCTGC

*Casp11* (murine *Casp4*) forward primer: GCTGATGCTGTCAAGCTGA

*Casp11* (murine *Casp4*) reverse primer: GTCTCGGTAGGACAAGTGATGTG

*Casp11* (murine *Casp4*) probe: CACGTGGAGAAGGACTTCATTGCC

Quantitative PCR was performed on magnetically enriched HSCs via negative selection using biotinylated CD5, CD19, CD11b, GR1, Ter119 (BioLegend) then magnetized streptavidin (Miltenyi) and using primer and FAM-MGB probe sets ordered from ThermoFisher Scientific: *Pdgfrb* (Mm00435553_m1), *Notch1* (Mm00627185_m1), *Ksr1* (Mm00516401_m1), *Syngap1* (Mm01306145_m1), *Abl1* (Mm00802029_m1), *Rasal1* (Mm00443428_m1), *Tiam1* (Mm00437079_m1), Trio (Mm01157671_m1), *Cbl* (Mm00483069_m1), *Rhob* (Mm00455902_s1).

### CyTOF

All mass cytometry reagents were purchased from Fluidigm Inc. (former DVS) unless otherwise noted. Mouse BM cell suspensions were washed with PBS containing 0.1% BSA and blocked with commercial Fc-blocking reagent (BD Bioscience) to minimize non-specific antibody binding. Cells were stained with metal-labeled or fluorochrome-labeled antibodies against cell surface markers (Supplementary Table 1) for 30 minutes on ice, washed and labeled with metal-labeled anti-fluorochrome antibodies. All antibodies were purchased pre-conjugated to metal tags, or conjugated in-house using MaxPar X8 conjugation kits. After antibody staining, cells were incubated with cisplatin for 5 minutes as a viability dye. The cells were then washed in PBS containing 1.6% formaldehyde and a 1:1000 dilution of IdU to label nucleated cells. Where indicated, intracellular CD19 staining was performed at this step. Immediately prior to acquisition, cells were washed in PBS, then in diH_2_0 and resuspended in diH_2_0 containing a 1:10 dilution of EQ 4 Element Calibration beads. After routine instrument tuning and optimization, samples were acquired on a CyTOF2 Mass Cytometer in sequential 10-minute acquisitions at an acquisition rate of <500 events/s. Resulting FCS files were concatenated and normalized using a bead-based normalization algorithm in the CyTOF acquisition software and uploaded to Cytobank for analysis (http://www.cytobank.org/). FCS files were manually pre-gated on Ir193 DNA^+^CD45^+^ events, excluding dead cells, doublets and DNA-negative debris. Gated CD45^+^ population was then used for gating and clustering in all subsequent analyses including spanning-tree progression analysis of density-normalized events (SPADE)^24^, Sunburst^20^, viSNE^23^, and heatmaps. Additional heatmaps of averaged data were generated using heatmap.2 from gplots package in R. z-scores for relative %IdU are the standard deviations of each genotype’s sample value from the mean of all four genotype’s values for each cell population individually.

### Colony forming assays

For pre-B CFC assay, 2.4x10^5^ whole bone marrow cells in 500 μL complete RPMI, 10%FBS, were added to 3 mL of methylcellulose media containing IL-7 (R&D Systems, Cat# HSC009). For hematopoietic stem cell CFC assay, 10^4^ sorted LSK cells in 500 μL complete RPMI, 10% FBS, were added to 3 mL of methylcellulose complete media without Epo (R&D Systems, Cat# HSC008). 1 mL of these cell suspensions was plated in 35mmx10mm Falcon petri dishes (VWR, Cat#351008), and all were incubated at 37^o^C for 8 days before colonies were counted. Recombinant IL-1β (Peprotech) and IL-18 (Peprotech) were added in RPMI media at time of colony seeding at a concentration of 1 ng/mL for both respectively.

### ImmGen

Relevant Immunological Genome Project (ImmGen) data was downloaded from www.immgen.org. Population names were preserved as annotated in the ImmGen database, except for BM resident macrophages and classical monocytes.

### Chimeras

Recipient mice were irradiated with 650rad twice. The following day, donor BM or fetal liver was harvested. Fetal liver and BM cells were mixed in a 1:9 ratio and 2x10^6^ total purified cells were subsequently intravenously injected into recipient mice. Because of the large ratio of *μ*MT to *Eμ-myc* BM cells, 90% or greater of resulting immune reconstitution is of *μ*MT background. Only the B cell compartment is filled by rapidly proliferating *Eμ-myc* cells. Resulting chimeras were thereafter checked every other day for disease progression and humanely sacrificed when either their tumors surpassed 1cm^3^ or their BCS fell below 2.

In other stroma chimera experiments, wild-type C57BL/6 and *Casp1*^*−/−*^, *Casp11*^*−/−*^, *Pycard*^*−/−*^, *Nlrp3*^*−/−*^, *Gsdmd*^*−/−*^, *Casp1*^*−/−*^*Casp11*^*−/−*^ CD45.2 recipient mice were irradiated with 950rad once. The following day, 5x10^6^ total donor bone marrow from sex and age matched wild type CD45.1 mice were harvested and transferred intravenously into irradiated recipients. Resulting chimeras were monitored weekly and femur/tibia BM evaluated at 10 weeks post reconstitution.

### LSK HSC cell sorting by flow cytometry

Total BM cells were extracted from femurs and tibias and the red blood cells removed with RBC lysis buffer (Sigma-Aldrich). For specific LSK isolation, we used a FACS Aria II (BD bioscience) with Lineage negative cocktail (clones: 145-2C11, RB6-8C5, M1/70, RA3-6B2, Ter-119, Biolegend), c-kit (clone 2B8, BD Pharmingen) and Sca-1 (clone D7, Biolegend) antibodies.

### RNA sequencing and analysis

RNA sequencing of sorted HSCs was performed by the Genomics Core of Weill Cornell Medicine on an Illumina NovaSeq 6000 instrument. Raw sequence reads were mapped to the mouse genome mm10 using STAR aligner version 2.5.3 (Ref. ^53^). Reads mapped to each gene were counted using Rsubread^54^. Prior to each further analysis, genes were prefiltered to remove lowly-expressed genes, retaining genes with 50 or more reads in at least 2 samples. Immunoglobulin genes were excluded from the analysis. X and Y-linked genes were also excluded since these would spuriously appear to be differentially expressed due to an imbalance of male and female mice in the genotype groups. Further analysis was performed using the DESeq2 R package version 1.22.2 (Ref. ^55^). Principal components analysis was performed on the 500 genes with highest variance after applying the DESeq2 variance stabilization transformation. Differential expression analysis was performed using the DESeq2 Wald test, with the resulting p values adjusted using the method of Benjamini and Hochberg.^56^ Significance was determined using a False Discovery Rate (FDR) threshold of 10%. Gene Ontology (GO) enrichment analysis was performed on sets of differentially expressed genes using the compareCluster function of the clusterProfiler R package^54^ version 3.14. Enrichment results were pruned to remove highly redundant GO terms using the clusterProfiler function simplify using a cutoff value of 0.7. Enrichment p values were corrected using the method of Benjamini and Hochberg.

### *Ex vivo* analysis by cytokine array, *kRas or caspase inhibitor treated coculture*, ELISA, and flow cytometry

Age-matched (4-6-week-old) *Casp1*^*−/−*^*Casp11*^*−/−*^ and C57BL/6J mice were sacrificed humanely. Femurs and tibias were isolated removing all muscle tissue and BM was flushed with 200 μL of DMEM with 20% FBS + 1X L-glutamine & 1X Pen/Strep per bone. Flushed bone marrow from each mouse was supplied with a clean tibia bone with a sagittal cut to open access to the bone stroma, then was incubated at 37^o^C for 6 h. The samples were then spun down (13,400 x g), supernatant collected, and all genotypically identical samples pooled together.

#### Abcam 96-cytokine array (ab193659)

Samples were diluted 1:1 with blocking buffer provided with the kit. Quantitative analysis of signal on membranes was performed using the ImageJ v1.53 software (NIH, USA).

#### Ex vivo BM coculture with RAS inhibitor treated HSC and caspase inhibitor treated stroma

BM from *Casp1*^*−/−*^*Casp11*^*−/−*^ and C57BL/6J wild-type mice were magnetically separated into CD45^–^ and CD45^+^ fractions using CD45 microbeads (Miltenyi). CD45^–^ fraction (stroma) underwent RBC lysis and was then treated with or without 1μM of pan caspase inhibitor Q-VD-OPH hydrate (AdooQ Bioscience) for 2 h at 37^o^C. CD45^+^ fraction was enriched for HSCs using the Direct Lineage Cell Depletion Kit (Miltenyi) and resulting HSCs were treated with the kRAS switch I/II pocket Inhibitor BI-2852 or the negative control BI-2853 (opnMe - Boehringer Ingelheim) for 3.5 h at 37^o^C. Treated HSCs were then coated with Cell Trace Violet Dye before coculturing with inhibitor-treated stroma at 1:2 ratio. Coculture fractions were evaluated at 8 h and 16 h.

#### Ex Vivo Transwell HSC - Stroma Migration Assay

BM from *Casp1*^*−/−*^*Casp11*^*−/−*^ and C57BL/6J wild-type mice were magnetically separated twice consecutively into CD45^–^ and CD45^+^ fractions using CD45 microbeads (Miltenyi). CD45^–^ fraction (stroma) was treated with or without 1μM of pan caspase inhibitor Q-VD-OPH hydrate (AdooQ Bioscience) for 2 h at 37^o^C. CD45^+^ fraction was enriched for HSCs using Direct Lineage Cell Depletion Kit (Miltenyi) and resulting HSCs treated with the kRAS switch I/II pocket Inhibitor BI-2852 or negative control BI-2853 (opnMe - Boehringer Ingelheim) for 3.5 h at 37^o^C. Rasi treated HSCs were seeded in upper insert of transwell with QVD-OPH-treated stroma seeded in lower base well of transwells at a 1:2 ratio, respectively. Transwell chamber setup was a 24-well with high position upper insert of over 5.5mm distance from well base and with pore size of 3μM. Separate HSC and stroma fractions in both upper insert and lower base were evaluated at 8, 20 and 24 h.

#### Flow cytometry for proliferation, surface cytokine receptors

Proliferating HSCs and stromal cells from BM enriched coculture were stained with the following antibodies: [BioLegend] > Lin- FITC (Clone: 145-2C11, RA3-6B2, M1/70, TER-119, RB6-8C5), CD45.1 BV510 (Clone: A20), CD45 BV650 (Clone:30-F11), Sca-1 BV711 (Clone:D7), c-Kit PeCy7 (Clone: 2B8), CCR1 APC/Fire750 (Clone: S15040E); [Thermofisher] > CD48 PerCpe710 (Clone: HM48-1), CCR5 PE (Clone: HM-7A4), CD120b primary antibody (Clone: TR75-54), Goat Armenian Hamster IgG (+L) Highly Cross-Adsorbed secondary antibody AF594, Blue reactive Viability dye (Indo-1 Blue); [BS Biosciences/Horizon] > CD45.2 BUV737 (Clone: 104), CXCR2 BV421 (Clone: V48-2310); [Abcam] > TNFRI FITC (Clone: 55R-170), TNFR II APC (Clone: HM102). All flow cytometry generated data on the Fortessa II Custom Order 5-laser system was analyzed using FlowJo x10.8.1 software and Proliferation dye indexing flow data was generated using the FlowJo v10 Biology Algorithm for Proliferation Modeling.

#### ELISA for cytokine and soluble receptors

Supernatant collected at 6 h (baseline), 8 h, and 16 h of BM baseline culture or enriched coculture was diluted 1:1 with RPMI media and DuoSet ELISA kits for assessment of mouse MIP-1alpha (CCL3), MIP1-gamma (CCL9/10) and MIP2 (CXCL2) (Bio-techne R&D systems DY450/463/452) levels. ELISAMax Deluxe mouse TNF kit (BioLegend - 430915) and SimpleStep mouse sTNFRI (TNFRSF1A) and sTNFRII (TNFRSF1B) ELISA Kits (Abcam – ab202412/202408) were also used to measure cytokine and soluble receptor levels. All plates were shaken and read at OD 450nm, and measured absorbance values used to calculate cytokine concentration in pico range per extrapolation via individual standard curve for each cytokine in Excel v16.7.

### Cytometric bead array

Bone marrow supernatant was collected by flushing both femurs and tibia with a total of 400μl of RPMI 10% FBS, followed by incubation for 5-6 hours at 37^o^C. Samples were centrifuged at 300 x g for 5 minutes to remove cellular debris, and undiluted supernatant was harvested. Cytokines were quantified in this supernatant using cytometric bead array (CBA) mouse inflammation kit (BD, 552364) and IL-1β CBA flex set (BD, 560232).

### Analysis of publicly available scRNA-Seq data for validation

Raw scRNA-Seq data for osteoblast, perivascular, and vascular bone marrow niche cells from Tikhonova A.N. *et al*.^38^ was downloaded from the NCBI GEO database (accession GSE108892, samples GSM2915575, GSM2915576, GSM2915577, GSM2915579, GSM3494769, and GSM3494771) in the form of gene expression count matrices. Processing and analysis were performed using Seurat^57^ version 5.0 and R version 4.3.1. Cells were filtered to retain only those with at least 600 and at most 5000 genes detected, at most 30,000 reads, at most 10% mitochondrial gene counts, and zero expression of Ptprc (CD45). Samples were processed and integrated using the standard Seurat workflow: FindVariableFeatures, ScaleData, RunPCA, and IntegrateLayers using the RPCAIntegration method and otherwise default parameters. Cell clusters were calculated using FindNeighbors, RunUMAP, and FindClusters on the first 30 PCs of the integrated rPCA, at resolutions of 0.6 through 3.0 in intervals of 0.2. A resolution of 1.0 was selected to use based on visual examination of a clustree plot (clustree R package version 0.5.1)^58^. Resulting clusters were annotated based on visual inspection of the normalized expression of known stromal cell type marker genes.

Raw scRNA-Seq data for bone marrow derived macrophage cells from McCarty E. *et al*.^59^ was downloaded from the NCBI GEO database (accession GSE229311, sample GSE229311) in the form of gene expression count matrices and corresponding barcode and feature files, which were imported using the Read10X function. Cells were filtered to retain only those with at least 200 and at most 2000 genes detected, and at most 1% mitochondrial gene counts. Expression was normalized using the NormalizeData function with default parameters (log1p normalization, scale factor 10,000).

Visualizations were generated using the Seurat functions DimPlot, FeaturePlot, and DotPlot, and the CellChat function StackedVlnPlot (CellChat R package, version 2.1.2)^60^.

#### Manual stromal cell type Seurat cluster annotation

We based our BM stromal cell type annotations on three markers: vasculature *VEcad* (*Cdh5*), mesenchymal stromal cell leptin-receptor (*Lepr*), and osteoblast *Col1a1*, used by Tikhonova A.N. *et al*.^38^ to sort major stromal cells shown to support HSC, including LEPR^+^ mesenchymal stromal cells, vascular endothelial cells^39,40^, and osteoblasts^41,42^. We further incorporated sub-markers based on two sources:

1) Classifications by Tikhonova A.N. *et al*.^38^:
  - *VEcad*^+^ cells annotated as bone marrow endothelial cells (BMEC) sub-marked into either arteriolar V1 *Ly6a*^*high*^ or sinusoidal V2 *Stab2*^*high*^ and indicated as LEPR^+^ when applicable for Seurat clusters 6 and 15.
  - *Lepr*^+^ cells annotated broadly as mesenchymal stromal cell (lepRMSC) and sub-marked as O1, O2, O3 based on low co-expression of *Col1a1* or V1, V2 based on low co-expression of *VEcad. Lepr*^+^ cell sub-markers for the four major clusters P1 *Mgp*^*high*^, P2 *Lpl*^*high*^, P3 *Wif1*^*high*^, and P4 *Spp1*^*high*^*Ibsp*^*high*^ were not used due to shared expression of most across the LepRMSC Seurat clusters.
  - *Col1a1*^+^ cells annotated as osteoblasts and further sub-marked for the three osteo-lineage. subpopulations as O1 (*Col16a1*^*high*^*Tnn*^*high*^), O2 (O2 *Fbn1*^*high*^*Igf1*^*high*^) and O3 (O3 *Bglap*^*high*^*Car3*^*high*^).

2) Associations with expression of each major marker and cluster location on UMAP plots.

*VEcad*^*+*^ BMEC: *Pecam1, Kdr, Emcn, Flt4*, and *Tek*

*Lepr*^*+*^ LepRMSC: *Adipog, Cxcl12, Kitl, Angpt1*, and *Grem1*

*Col1a1*^*+*^ Osteoblast: *Bglap, Runx2, Sp7, Alpl, Spp1, Pth1r, Mmp13, Grem1, Sox9*, and *Cxcl12*

### Statistical approaches

No statistical methods were used to pre-determine sample sizes, but our sample sizes are similar to those reported in previous publications^61,62,63,64,65^. Data collection and analysis were not performed blind to the conditions of the experiments; only objective readouts were used to avoid user-dependent influence. Mice were assigned to experimental groups based on their genotypes. Thus, no further randomization was possible. Mice were co-housed to avoid introduction of any potential environmental covariates. No animals or data points were excluded from the analyses. Data distribution was assumed to be normal but this was not formally tested. Except for survival, all significance values were determined by 2-Way ANOVA, or 1-Way ANOVA with Dunnett’s, Tukey’s or Šídák’s multi-comparison post-test or unpaired two-tailed Student’s t-tests using Prism v9.5.1 and v10.3.1 software. Kaplan-Meier curve significance was determined by Log rank (Mantel-Cox) test using Prism v9.5.1 software.

## Extended Data

**Extended Data Figure 10:**
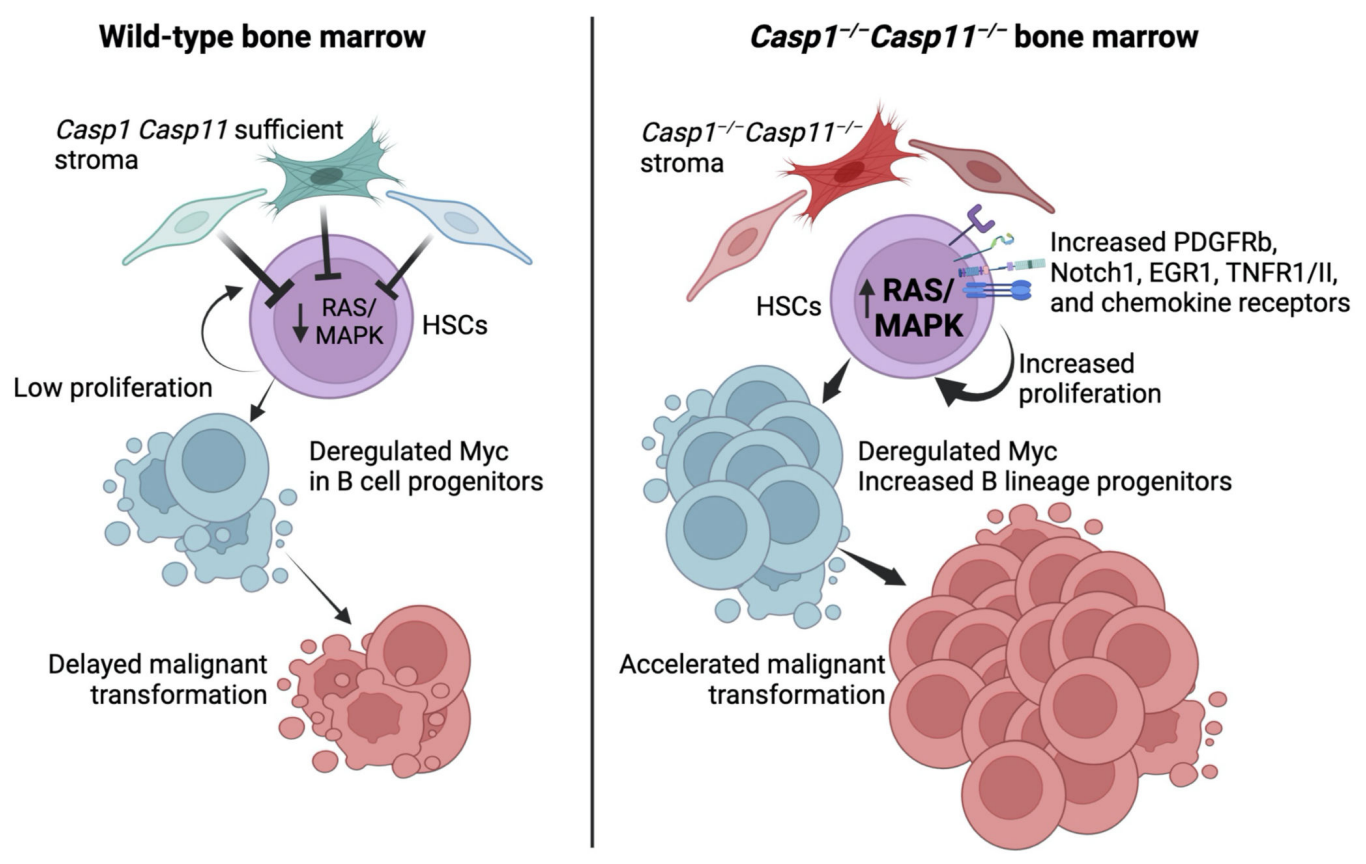


**Extended Data Table 1:** 

Primary Antibody	Secondary antibody	Clone	Vendor	Catalog number	Metal ion conjugate
Ter119		Ter119	Biolegend	116201	115 In
Ly6G		HK1.4	Biolegend	127601	141 Pr
caspase3		D3E9	DVS	3142004A	142 Nd
CD41		MWreg30	DVS	3143009B	143 Nd
CD24		M1/69	Biolegend	101801	144 Nd
BP1-PE		6C3	eBioscience	12-5891-81	
	anti-PE	PE001	DVS	3145006B	145 Nd
F4/80		BM8	DVS	3146008B	146 Nd
CD45		30-F11	DVS	3147003B	147 Sm
CD11b		M1/70	DVS	3148003B	148 Nd
CD19		6D5	DVS	3149002B	149 Sm
IgD		11-26c.2a	DVS	3150011B	150 Nd
IgM		RMM-1	DVS	3151006B	151 Eu
Siglec F		E50-2440	BD	552125	152 Sm
PDCA-1		927	Biolegend	127002	153 Eu
CD48		HM48-1	DVS	3154004B	154 Sm
CD14		Sa14-2	DVS	3156009B	156 Gd
pSTAT3		Y705	DVS	3158005A	158 Gd
IL-6R		D7715A7	Biolegend	115807	159 Tb
GL-7-FITC		GL-7	Biolegend	144603	
	anti-FITC	FIT-22	DVS	3160011B	160 Gd
Ly6C		HK1.4	Biolegend	128001	162 Dy
CD43-APC		S7	BD	BD560663	
	anti-APC	APC003	DVS	3163001B	163 Dy
Sca-1		D7	DVS	3164005B	164 Dy
CD127		A7R34	Biolegend	135002	165 Ho
cKit		SB8	DVS	3166004B	166 Er
CD150		TC15	DVS	3167004B	167 Er
CD123		5B11	Biolegend	106002	169 Tm
CD34-biotin		HM34	Biolegend	128603	
	anti-biotin	ID4-C5	DVS	3170003B	170 Er
CD38		90	DVS	3171007B	171 Yb
MHC II		M5/144	DVS	9174003B	174 Yb
Flt3		A2F10	eBioscience	14-1351-82	175 Lu
B220		RA3-6B2	DVS	3176002B	176 Yb
Thy1.2 efluorNC 650		53-2.1	eBioscience	95-0902-42	110-116Cd
					
**Non-Antibody Signals**					
Cisplatin					195 Pt
IdU					

CyTOF antibody and stain list.

## Supplementary Material

Supplementary Data 1

Supplementary Data 2

Supplementary Table 1

## Data Availability

Source Data files for all data presented are available through Nature Immunology.

## Code Availability

The RNAseq data have been uploaded to the Gene Expression Omnibus (GEO) under accession GSE183695. The code for data processing and analysis in bioinformatics is fully referenced and described in Methods.
