# Supplementary material for: A stromal inflammasome Ras-safeguard against Myc driven lymphomagenesis
Source: Nat Immunol. Author manuscript; Available in PMC 2025 Nov 17. (PMC7618359; doi:10.1038/s41590-024-02028-z)
Supplement: Supplementary Table 1 [file EMS210572-supplement-Supplementary_Table_1.pdf]

nature immunology

Supplementary information https://doi.org/10.1038/s41590-024-02028-z

A stromal inflammasome Ras safeguard against Myc-driven lymphomagenesis

In the format provided by the authors and unedited

Primary Antibody	Secondary antibody	Clone	Vendor	Catalog number	Metal ion conjugate
Ter119		Ter119	Biolegend	116201	115 In
Ly6G		HK1.4	Biolegend	127601	141 Pr
caspase3		D3E9	DVS	3142004A	142 Nd
CD41		MWreg30	DVS	3143009B	143 Nd
CD24		M1/69	Biolegend	101801	144 Nd
BP1-PE		6C3	eBioscience	12-5891-81	
	anti-PE	PE001	DVS	3145006B	145 Nd
F4/80		BM8	DVS	3146008B	146 Nd
CD45		30-F11	DVS	3147003B	147 Sm
CD11b		M1/70	DVS	3148003B	148 Nd
CD19		6D5	DVS	3149002B	149 Sm
IgD		11-26c.2a	DVS	3150011B	150 Nd
IgM		RMM-1	DVS	3151006B	151 Eu
Siglec F		E50-2440	BD	552125	152 Sm
PDCA-1		927	Biolegend	127002	153 Eu
CD48		HM48-1	DVS	3154004B	154 Sm
CD14		Sa14-2	DVS	3156009B	156 Gd
pSTAT3		Y705	DVS	3158005A	158 Gd
IL-6R		D7715A7	Biolegend	115807	159 Tb
GL-7-FITC		GL-7	Biolegend	144603	
	anti-FITC	FIT-22	DVS	3160011B	160 Gd
Ly6C		HK1.4	Biolegend	128001	162 Dy
CD43-APC		S7	BD	BD560663	
	anti-APC	APC003	DVS	3163001B	163 Dy
Sca-1		D7	DVS	3164005B	164 Dy
CD127		A7R34	Biolegend	135002	165 Ho
cKit		SB8	DVS	3166004B	166 Er
CD150		TC15	DVS	3167004B	167 Er
CD123		5B11	Biolegend	106002	169 Tm
CD34-biotin		HM34	Biolegend	128603	
	anti-biotin	ID4-C5	DVS	3170003B	170 Er
CD38		90	DVS	3171007B	171 Yb
MHC II		M5/144	DVS	9174003B	174 Yb
Flt3		A2F10	eBioscience	14-1351-82	175 Lu
B220		RA3-6B2	DVS	3176002B	176 Yb
Thy1.2 efluorNC 650		53-2.1	eBioscience	95-0902-42	110-116Cd
**Non-Antibody Signals**					
Cisplatin					195 Pt
IdU					

CyTOF antibody and stain list

Extended Data Table 1
